# Piezo1 expression in chondrocytes controls endochondral ossification and osteoarthritis development

**DOI:** 10.1038/s41413-024-00315-x

**Published:** 2024-02-23

**Authors:** Laura J. Brylka, Assil-Ramin Alimy, Miriam E. A. Tschaffon-Müller, Shan Jiang, Tobias Malte Ballhause, Anke Baranowsky, Simon von Kroge, Julian Delsmann, Eva Pawlus, Kian Eghbalian, Klaus Püschel, Astrid Schoppa, Melanie Haffner-Luntzer, David J. Beech, Frank Timo Beil, Michael Amling, Johannes Keller, Anita Ignatius, Timur A. Yorgan, Tim Rolvien, Thorsten Schinke

**Affiliations:** 1 Department of Osteology and Biomechanics, University Medical Center Hamburg-Eppendorf, 20246 Hamburg, Germany; 2 Department of Trauma and Orthopedic Surgery, University Medical Center Hamburg-Eppendorf, 20246 Hamburg, Germany; 3 Institute of Orthopedic Research and Biomechanics, University Medical Center Ulm, Baden-Württemberg, 89081 Ulm, Germany; 4 Department Legal Medicine, University Medical Center Hamburg-Eppendorf, 20246 Hamburg, Germany; 5 Leeds Institute of Cardiovascular and Metabolic Medicine, School of Medicine, University of Leeds, LS2 9JT Leeds, UK

**Keywords:** Diseases, Physiology

## Abstract

Piezo proteins are mechanically activated ion channels, which are required for mechanosensing functions in a variety of cell types. While we and others have previously demonstrated that the expression of *Piezo1* in osteoblast lineage cells is essential for bone-anabolic processes, there was only suggestive evidence indicating a role of Piezo1 and/or Piezo2 in cartilage. Here we addressed the question if and how chondrocyte expression of the mechanosensitive proteins Piezo1 or Piezo2 controls physiological endochondral ossification and pathological osteoarthritis (OA) development. Mice with chondrocyte-specific inactivation of Piezo1 (*Piezo1*^*Col2a1Cre*^), but not of Piezo2, developed a near absence of trabecular bone below the chondrogenic growth plate postnatally. Moreover, all *Piezo1*^*Col2a1Cre*^ animals displayed multiple fractures of rib bones at 7 days of age, which were located close to the growth plates. While skeletal growth was only mildly affected in these mice, OA pathologies were markedly less pronounced compared to littermate controls at 60 weeks of age. Likewise, when OA was induced by anterior cruciate ligament transection, only the chondrocyte inactivation of Piezo1, not of Piezo2, resulted in attenuated articular cartilage degeneration. Importantly, osteophyte formation and maturation were also reduced in *Piezo1*^*Col2a1Cre*^ mice. We further observed increased Piezo1 protein abundance in cartilaginous zones of human osteophytes. Finally, we identified *Ptgs2* and *Ccn2* as potentially relevant Piezo1 downstream genes in chondrocytes. Collectively, our data do not only demonstrate that Piezo1 is a critical regulator of physiological and pathological endochondral ossification processes, but also suggest that Piezo1 antagonists may be established as a novel approach to limit osteophyte formation in OA.

## Introduction

Cellular mechanotransduction is of fundamental importance to control tissue homeostasis, and the skeleton is one prime example for the relevance of mechanically induced physiological and pathological processes.^1–3^ In general, the cellular response to various mechanical triggers, including fluid flow, hydrostatic pressure, cellular overcrowding or matrix rigidity, can be sensed by specific transmembrane proteins, among which the two members of the Piezo family play a prominent role.^4,5^ More specifically, Piezo1 and Piezo2 are large transmembrane ion channels that are activated by mechanical stimulation.^6–8^ They were found to be of crucial importance for mechanosensation in a variety of different cell types and have thereby gained considerable attention in the scientific community.^4,9–12^ This culminated in awarding the 2021 Nobel Prize in Physiology or Medicine to Ardem Patapoutian, whose ground-breaking studies led to the identification of the Piezo channels in 2010.^6,13^ Besides demonstrating that these proteins are activated by mechanical stimulation, his group also identified a small synthetic molecule termed Yoda1 as a Piezo1-specific agonist.^14^ On the other hand, one possibility to inhibit Piezo proteins is to utilize the spider venom peptide termed GsMTx4, albeit its antagonistic action is not specific to Piezo1, as it also inhibits other cationic mechanosensitive channels.^15–17^

Skeletal development, growth and maintenance are complex processes involving several unique cell types, whose functional interactions are regulated by many different mechanisms, involving mechanical cues.^18–20^ Recent studies, mostly obtained by bone phenotyping of conditional knockout mouse models, have established the critical role of Piezo1 as a mechanosensitive transmembrane protein in osteoblast lineage cells.^5,21–24^ More specifically, by using *Piezo1*^*fl/fl*^ mice and different *Cre* drivers for Piezo1 inactivation at various stages of osteoblast differentiation, it was found that Piezo1 is essential to promote skeletal development, bone mass acquisition, the crosstalk with bone-resorbing osteoclasts, as well as the osteocyte-dependent osteoanabolic response towards mechanical loading. Whereas the latter function was also supported by our own analysis of *Piezo1*^*Dmp1Cre*^ mice, we additionally identified an unexpected phenotype in *Piezo1*^*Runx2Cre*^ mice, where trabecular bone structures below the chondrogenic growth plates were essentially absent.^25^ Since *Runx2*, encoding a transcription factor required for osteoblast differentiation, is also expressed in growth plate chondrocytes,^26,27^ these results suggested that *Piezo1* expression in chondrocytes is required for their transdifferentiation into bone-forming osteoblasts.^18,28^ This hypothesis was subsequently confirmed, not only by the persistent expression of type X collagen in *Piezo1*^*Runx2Cre*^ subchondral bone cells, but also by an initial analysis of 12-week-old *Piezo1*^*Col2a1Cre*^ mice, which displayed a striking reduction of trabecular bone mass without affection of cortical bone.^25^

With respect to articular chondrocytes, it was reported that both Piezo proteins synergize to mediate mechanosensing properties in vitro and in an explant-cartilage injury model.^29^ Moreover, a recent study demonstrated that the severity of surgically induced osteoarthritis (OA) in mice is reduced by intraarticular injection of GsMTx4.^30^ At the time we were preparing our manuscript, two publications have reported the analysis of surgically induced OA progression in mice with conditional inactivation of Piezo proteins in chondrocytes. The first study utilized *Gdf5*^*Cre*^ transgenic mice to inactivate Piezo1 and Piezo2, but the respective mice did not differ from *Cre*-negative controls in terms of OA progression after destabilization of the medial meniscus.^31^ The second study used an inducible system (*Acan*^*CreERT2*^*)* to selectively inactivate Piezo1, and these mice displayed a significant attenuation of OA development, whereas intra-articular injection of Yoda1 aggravated the surgically induced OA pathologies.^32^ Since OA is a debilitating, highly prevalent joint disease affecting over 500 million people worldwide,^33^ novel disease-modifying therapies are of great interest. It was therefore important to address the question, if and how Piezo1 and/or Piezo2 are involved in OA development, especially since the two manuscripts mentioned above^31,32^ came to entirely different conclusions.

In the present study we utilized the *Col2a1*^*Cre*^ transgenic mouse line to generate animals with chondrocyte-specific inactivation of the *Piezo* genes for in-depth skeletal phenotyping and response to surgically induced OA. Our findings provide evidence that chondrocyte expression of Piezo1, but not of Piezo2, is not only relevant for postnatal trabecular bone formation but also for cartilage degeneration and osteophyte formation in surgically induced or age-related OA. This suggests that targeting Piezo1 with specific antagonists may represent a promising therapeutic approach to limit OA development and progression in patients.

## Results

### Chondrocyte expression of Piezo1 is physiologically required for postnatal trabecular bone formation below the growth plate

**Fig. 1:**
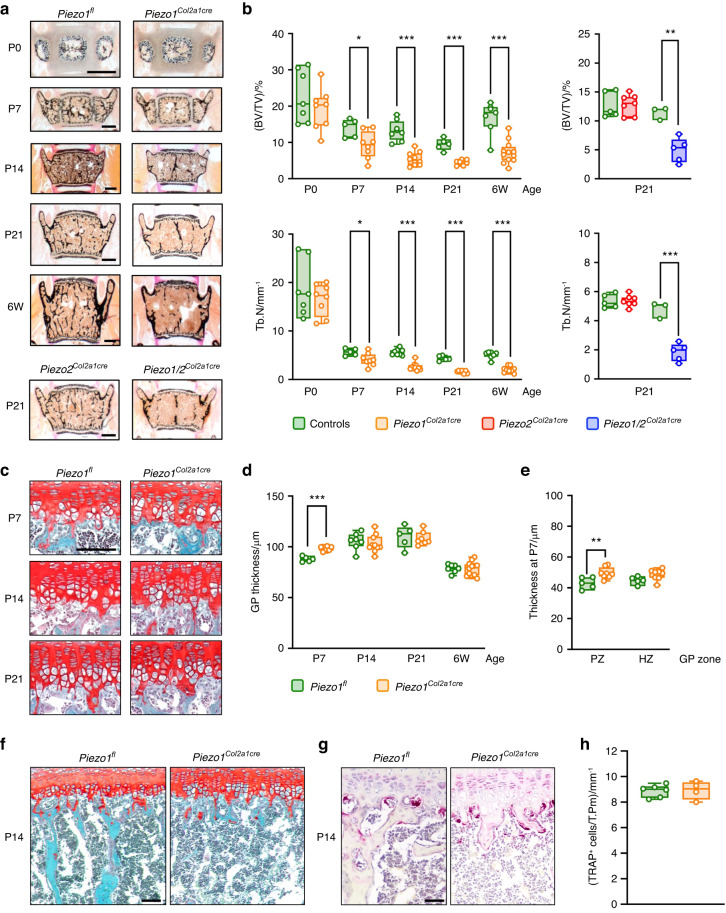
Inactivation of *Piezo1* in chondrocytes impairs postnatal trabecular bone formation. **a** Representative von Kossa-stained sections of vertebral bodies from *Piezo1*^*fl*^ and *Piezo1*^*Col2a1Cre*^ littermates immediately after birth (P0), at postnatal days 7, 14 and 21 (P7, P14, P21) or at 6 weeks of age (6 W). Mineralized bone is stained in black. Scale bars: 500 µm. Bottom images show representative sections of vertebral bodies from *Piezo2*^*Col2a1Cre*^ and *Piezo1/2*^*Col2a1Cre*^ mice at postnatal day 21. **b** Histomorphometric quantification of the trabecular bone volume per tissue volume (BV/TV) and the trabecular number (Tb.N) in mice of different ages and genotypes, as indicated (*n* ≥ 3). **c** Representative Safranin O-stained sections of the lumbar spine showing growth plates from *Piezo1*^*fl*^ and *Piezo1*^*Col2a1Cre*^ littermates at postnatal day 7, 14 and 21. Cartilage is stained in red. Scale bar: 100 µm. **d** Histomorphometric quantification of the growth plate (GP) thickness in mice of different ages and genotypes, as indicated (*n* ≥ 5). **e** Histomorphometric quantification of proliferative zone (PZ) and hypertrophic zone (HZ) thickness in mice at P7 (*n* ≥ 5). **f** Representative Safranin O-stained sections of the lumbar spine in mice at P14, demonstrating the absence of trabecular bone structures below the growth plate in *Piezo1*^*Col2a1Cre*^ mice. Scale bar: 250 µm. **g** Representative sections of the lumbar spine at the age of P14 stained for TRAP activity. Scale bar: 50 µm. **h** Quantification of the number of TRAP-positive cells per tissue perimeter (TRAP^+^ cells/T.Pm) below the growth plate (*n* ≥ 4). Statistical analysis was conducted with Student’s *t* test comparing different genotypes for each age. *P < 0.05, **P < 0.01, ***P < 0.001

To analyze the function of Piezo proteins in chondrocytes, we generated mice with conditional inactivation of Piezo1 and/or Piezo2 for in-depth characterization of their skeletal phenotype. Histomorphometric quantification performed on undecalcified vertebral body sections demonstrated that *Piezo1*^*Col2a1Cre*^ mice progressively develop a low trabecular bone mass phenotype with postnatal onset (Fig. 1a). When analyzing 3-week-old mice, we additionally observed that the extent of trabecular bone mass reduction was comparable between *Piezo1/2*^*Col2a1Cre*^ and *Piezo1*^*Col2a1Cre*^ mice, whereas *Piezo2*^*Col2a1Cre*^ mice did not show this pathology (Fig. 1b). In stark contrast to the severe reduction of trabecular bone mass, the impact of chondrocyte-specific Piezo1 inactivation on the growth plate was moderate (Fig. 1c). More specifically, whereas the proper alignment of chondrocytes was not disturbed, a significant widening of the growth plate was only observed at postnatal day P7 (Fig. 1d), which was attributable to an extension of the proliferative zone (Fig. 1e). At that age there was no growth impairment for *Piezo1*^*Col2a1Cre*^ mice observed, yet the length of the lumbar spine was significantly reduced at postnatal day P21 and thereafter (Fig. S1a). Importantly, however, there was no genotype-dependent difference of chondrocyte number per cartilage area, neither at P7, nor at P21 (Fig. S1b).

In contrast to the essentially unaffected chondrocyte population in *Piezo2*^*Col2a1Cre*^ mice, there was a strong reduction of the trabecular bone structures below the growth plates (Fig. 1f), which was also confirmed by immunohistochemical staining of type I and type II collagens (Fig. S1c). This phenotype was however not accompanied by an increased abundance of cells stained positive for activity of the osteoclast marker tartrate-resistant acid phosphatase (TRAP), indicating that it is not the consequence of excessive resorption (Fig. 1g, h). Since the near absence of trabecular bone in these areas prevented a comparative histomorphometric quantification, we focused on the remaining trabecular bone structures located in the center of the vertebral body sections from 6-week-old mice (Fig. S2a). Although we observed, similar to what we have previously described for the *Piezo1*^*Runx2Cre*^ model,^25^ that the osteoblasts in *Piezo2*^*Col2a1Cre*^ mice displayed an unusual flattened appearance, there were no significant differences in osteoblast and osteoclast indices between *Piezo1*^*Col2a1Cre*^ mice and *Piezo1*^*fl*^ control littermates (Fig. S2b, c). Taken together, these findings show that inactivation of Piezo1, but not of Piezo2, in chondrocytes only causes a moderate and transient growth plate pathology, and that the major function of Piezo1 is to enable postnatal trabecular bone formation in the process of endochondral ossification.

**Fig. 2:**
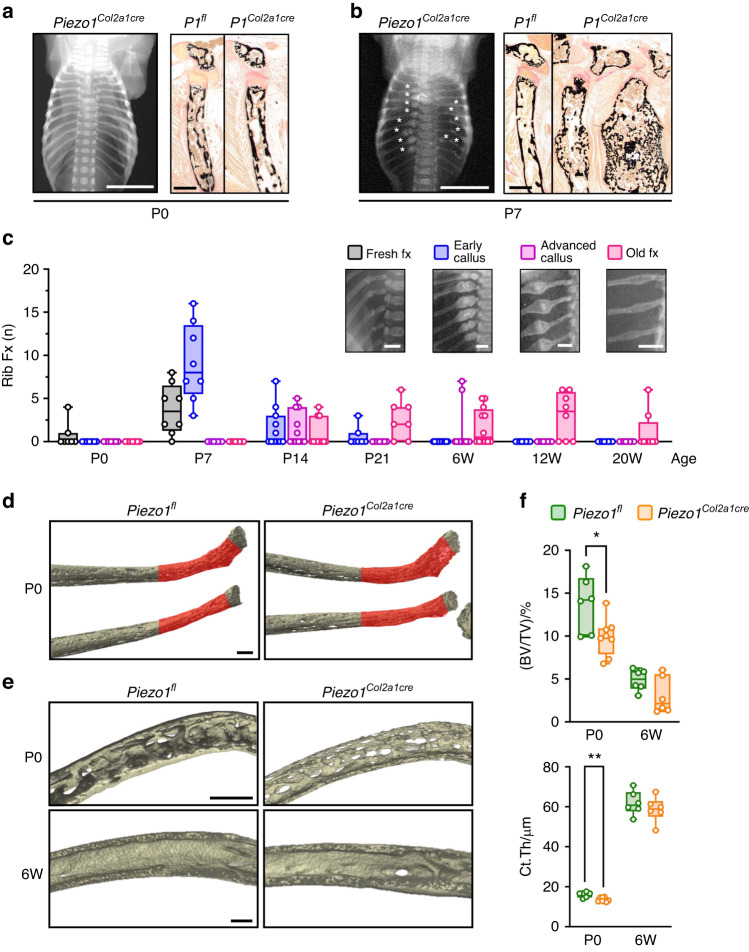
Inactivation of *Piezo1* in chondrocytes causes rib fractures in neonatal mice. Representative contact radiographs and histology images of rib bones from *Piezo1*^*fl*^ and *Piezo1*^*Col2a1Cre*^ littermates **a** immediately after birth (P0) or **b** at postnatal day 7 (P7), as indicated. Scale bar in radiographs: 5 mm, in histological sections: 250 µm. **c** Quantification of rib fractures in *Piezo1*^*Col2a1Cre*^ mice immediately after birth (P0), at postnatal days 7, 14 and 21 (P7, P14, P21) or at 6, 12 and 20 weeks of age (6 W, 12 W, 20 W). Scale bars: 2 mm. **d** Representative µCT images of rib bones from *Piezo1*^*fl*^ and *Piezo1*^*Col2a1Cre*^ littermates immediately after birth (P0), indicating the analyzed region of interest in red. Scale bar: 50 µm. **e** Representative close-up cross-sectioned µCT reconstructions of the quantified areas from *Piezo1*^*fl*^ and *Piezo1*^*Col2a1Cre*^ littermates at P0 and at 6 weeks of age (6 W). Scale bar: 100 µm. **f** Quantification of the trabecular bone volume per tissue volume (BV/TV) and the cortical thickness (Ct.Th) in mice with different ages and genotypes, as indicated. *n* ≥ 6. Statistical analysis was conducted with Student’s *t* test comparing different genotypes for each age. *P < 0.05, **P < 0.01

In our previous study we additionally identified a high incidence of rib fractures, not only in the *Piezo1*^*Runx2Cre*^ model, but also in a first set of *Piezo1*^*Col2a1Cre*^ mice at 2 and 12 weeks of age.^25^ Importantly, however, whereas we previously only documented this phenotype based on contact radiographs, we now performed additional studies to focus on this unique pathology, which included the analysis of *Piezo1*^*Col2a1Cre*^ mice at different ages. Undecalcified histology of rib bones did not only confirm the absence of fractures in the majority of newborn *Piezo1*^*Col2a1Cre*^ mice (Fig. 2a), but also indicated that the fracture calli found in rib bones of 1-week-old *Piezo1*^*Col2a1Cre*^ mice were located close to the growth plates (Fig. 2b). Moreover, in non-fractured ribs of these mice, we observed a similar pathology as found for the vertebral bodies, i.e. a reduced number of trabeculae below the hypertrophic zone (Fig. S3). The healing of the rib fractures explained why their incidence in *Piezo1*^*Col2a1Cre*^ mice declined after a peak at the age of one week (Fig. 2c). Importantly, all of the analyzed one-week-old *Piezo1*^*Col2a1Cre*^ mice displayed multiple rib bone fractures in the same regions (Fig. S4a). Since this phenotype excluded a quantitative analysis based on micro-computed tomography (µCT) scans at P7 (Fig. S4b), we utilized a high-resolution µCT system to focus our analysis on rib bones of newborn animals (Fig. S5). More specifically, we quantified trabecular and cortical bone parameters in the fracture-prone regions in rib bones of *Piezo1*^*fl*^ and *Piezo1*^*Col2a1Cre*^ littermates immediately after birth and at 6 weeks of age, a time point at which no new fractures occurred and old fractures were healed (Fig. 2d, e). Here we observed reduced trabecular bone mass and cortical thickness in newborn *Piezo1*^*Col2a1Cre*^ mice (Fig. 2f). On the other hand, trabecular bone structures in 6-week-old animals were strongly reduced independent of the genotype, yet the age-dependent increase in cortical bone thickness was not affected by chondrocyte inactivation of Piezo1. Together, these data demonstrate that *Piezo1* expression in chondrocytes is a prerequisite for the structural integrity of rib bones. Since the rib fracture phenotype affects all *Piezo1*^*Col2a1Cre*^ mice at a similar location close to the chondrogenic growth plates, and since it is preceded by reduced trabecular bone mass in these regions, the respective data provide further evidence for a major function of Piezo1 for endochondral ossification.

### Chondrocyte-specific inactivation of Piezo1 protects against primary OA

**Fig. 3:**
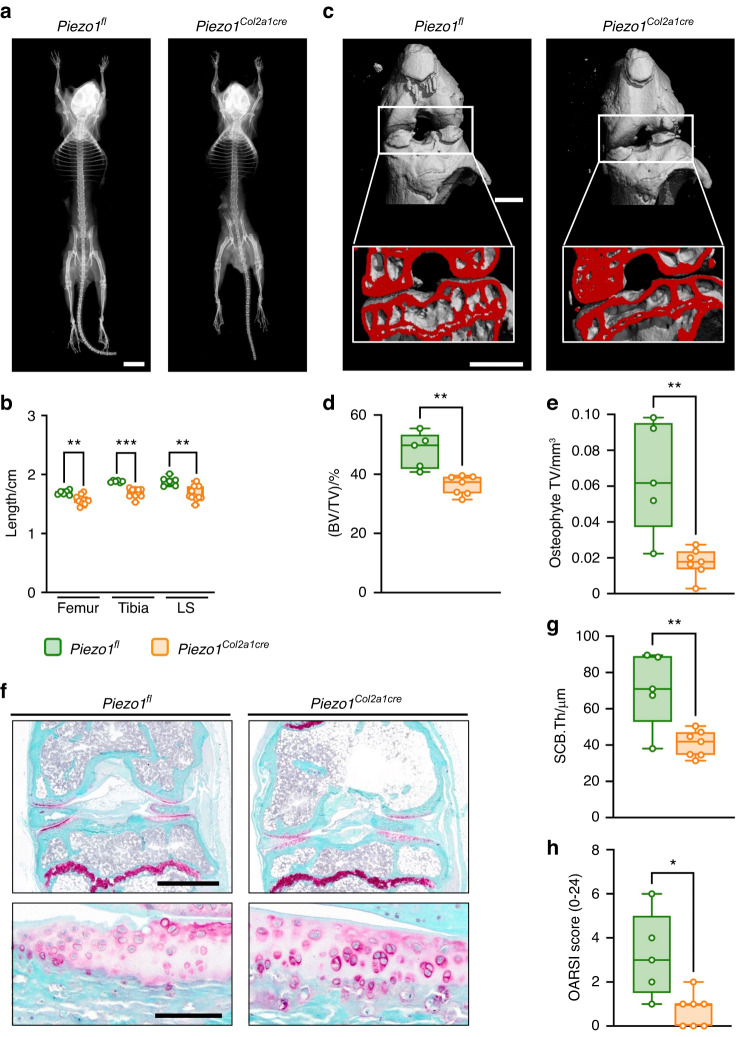
Inactivation of *Piezo1* in chondrocytes attenuates OA development in aged (60-week-old) mice. **a** Representative contact radiographs of *Piezo1*^*fl*^ and *Piezo1*^*Col2a1Cre*^ mice, demonstrating mildly reduced body length in *Piezo1*^*Col2a1Cre*^ mice. Scale bar: 10 mm **b** Quantification of the skeletal length of the femur, tibia, and lumbar spine (LS). **c** Representative µCT images of knee joints from *Piezo1*^*fl*^ and *Piezo1*^*Col2a1Cre*^ mice. Scale bars: 1 mm. **d** Quantification of subchondral bone volume fraction (BV/TV). **e** Quantification of the osteophyte total volume (TV). **f** Representative Safranin-O staining of undecalcified knee joint sections from *Piezo1*^*fl*^ and *Piezo1*^*Col2a1Cre*^ mice. Top panel: overview, scale bar: 1 mm. Bottom panel: detailed view of the articular cartilage, scale bar: 100 µm. **g** Histomorphometric quantification of the subchondral bone plate thickness (SCB.Th). **h** Quantification of the OARSI score in knee joints of 60-week-old *Piezo1*^*fl*^ and *Piezo1*^*Col2a1Cre*^ mice (*n* ≥ 5). Statistical analysis was conducted with Student’s *t* test. *P < 0.05, **P < 0.01, ***P < 0.001

The above-described healing of the rib fractures allowed us to study older *Piezo1*^*Col2a1Cre*^ mice, which did not display an obvious health burden. In fact, contact radiography revealed no apparent skeletal dysplasia (Fig. 3a), and only a slight length reduction of different skeletal sites was observed in 60-week-old *Piezo1*^*Col2a1Cre*^ mice (Fig. 3b). This enabled us to analyze them for a potential articular cartilage phenotype and to address the relevance of Piezo1 in primary OA development. By performing µCT of the knee joints, we identified several distinct differences between 60-week-old *Piezo1*^*fl*^ and *Piezo1*^*Col2a1Cre*^ mice (Fig. 3c). More specifically, not only the subchondral trabecular bone volume (Fig. 3d), but also the formation of osteophytes was reduced by Piezo1 inactivation in chondrocytes (Fig. 3e). We further applied undecalcified histology and found that age-associated OA development was less pronounced in *Piezo1*^*Col2a1Cre*^ mice (Fig. 3f). Compared with *Piezo1*^*fl*^ controls, the thickness of the subchondral bone plate was lower in *Piezo1*^*Col2a1Cre*^ mice (Fig. 3g). Most importantly, primary OA development, as quantified by the OARSI scoring system, was markedly mitigated in *Piezo1*^*Col2a1Cre*^ mice (Fig. 3h). These data suggest that *Piezo1* deficiency in chondrocytes protects against primary OA.

### Chondrocyte-specific inactivation of Piezo1 attenuates cartilage degeneration and osteophyte formation after surgical induction of OA

**Fig. 4:**
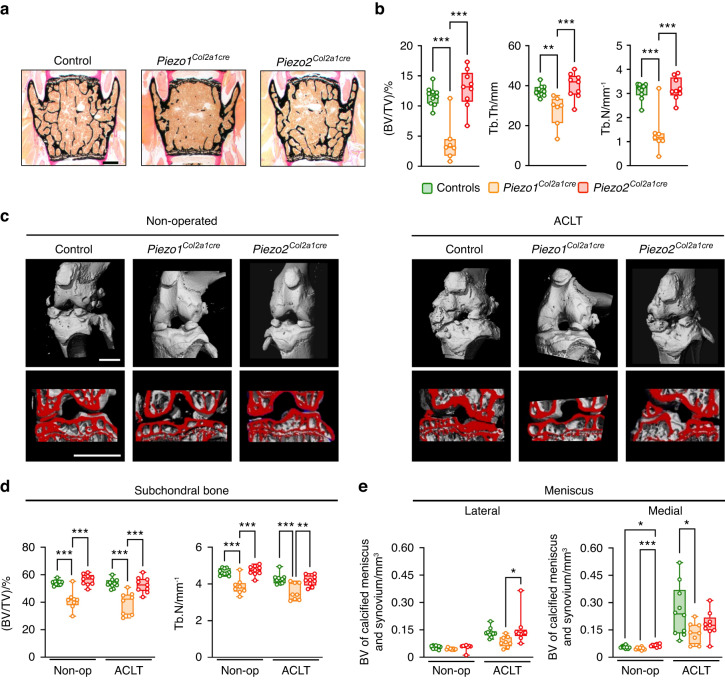
Inactivation of *Piezo1* in chondrocytes impairs trabecular bone mass and limits OA development after ACLT. **a** Representative von Kossa-stained sections of undecalcified vertebral bodies of control, *Piezo1*^*Col2a1cre*^, and *Piezo2*^*Col2a1Cre*^ mice. Scale bar: 500 µm. **b** Histomorphometric quantification of trabecular bone volume (BV/TV), trabecular thickness (Tb. Th), and trabecular number (Tb. N) (*n* ≥ 8). **c** Representative µCT images of knee joints from non-operated and ACLT control, *Piezo1*^*Col2a1cre*^, and *Piezo2*^*Col2a1Cre*^ mice showing reduced subchondral bone volume in non-operated and ACLT *Piezo1*^*Col2a1cre*^ but not *Piezo2*^*Col2a1Cre*^ mice, scale bars: 1 mm. **d** µCT-based quantification of subchondral bone volume fraction (BV/TV) and trabecular number (Tb.N). **e** µCT-based quantification of calcified meniscus and synovium assessed in the lateral and medial joint compartment of non-operated and ACLT control, *Piezo1*^*Col2a1cre*^, and *Piezo2*^*Col2a1Cre*^ mice

To further investigate the observed effect on OA development in the clinically relevant context of posttraumatic OA, we applied anterior cruciate ligament transection (ACLT) in order to induce OA in *Piezo1*^*Col2a1Cre*^, *Piezo2*^*Col2a1Cre*^, and their control littermate mice. Surgery was performed at the age of 12 weeks, and the phenotypic analysis was carried out 8 weeks thereafter. For histologic analysis of the operated 20-week-old mice, we first analyzed vertebral body sections (Fig. 4a). We again observed that only the *Piezo1*^*Col2a1Cre*^ mice displayed an almost complete absence of trabecular bone, which was mainly attributable to a reduction of the trabecular number (Fig. 4b). To assess OA pathologies, we next applied µCT scanning to visualize the non-operated and operated knee joints (Fig. 4c). The subsequent quantitative analysis revealed that the subchondral bone volume in the tibia was reduced in *Piezo1*^*Col2a1Cre*^, but not in *Piezo2*^*Col2a1Cre*^ mice, independent of the ACLT surgery (Fig. 4d). In addition, we detected increased meniscal and synovial calcification in the lateral and medial compartments of the knee joint after ACLT as another OA-related pathology (Fig. 4e), which was attenuated in *Piezo1*^*Col2a1Cre*^ but not in *Piezo2*^*Col2a1Cre*^ mice.

**Fig. 5:**
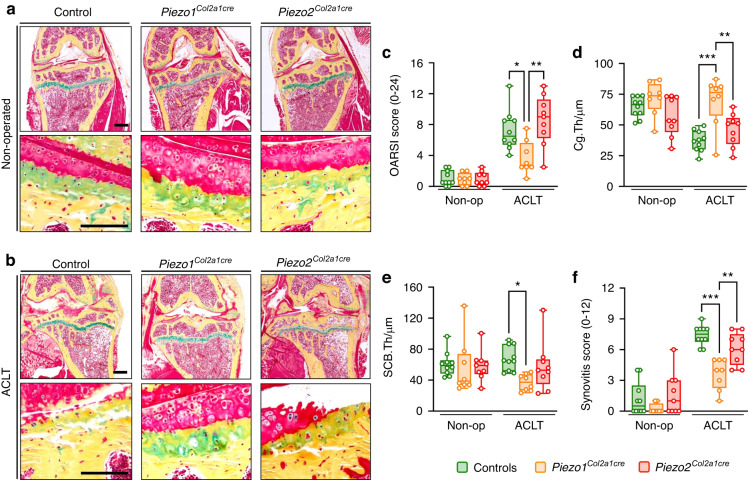
Inactivation of *Piezo1* in chondrocytes attenuates cartilage degradation and inflammation after ACLT. **a** Movat pentachrome-stained sections of undecalcified non-operated knee joints from control, *Piezo1*^*Col2a1cre*^, and *Piezo2*^*Col2a1Cre*^ mice. Top panel: overview, scale bar: 500 µm; bottom panel: detailed view of the osteochondral unit, scale bar: 100 µm. **b** Movat pentachrome-stained sections of undecalcified anterior cruciate ligament transected (ACLT) knee joints from control, *Piezo1*^*Col2a1cre*^, and *Piezo2*^*Col2a1Cre*^ mice. Top panel: overview, scale bar: 500 µm; bottom panel: detailed view of the osteochondral unit, scale bar: 100 µm. **c** Histomorphometric quantification of the OARSI score, **d** cartilage thickness (Cg. Th), **e** subchondral bone plate thickness (SCB. Th), and **f** synovitis score (*n* ≥ 8). Statistical analysis was conducted with one-way ANOVA (Tukey). *P < 0.05, **P < 0.01, ***P < 0.001

By applying cellular histomorphometry on histological sections from subchondral bone we found that ACLT caused a significant reduction of the osteoblast number per bone perimeter (Fig. S6a), whereas osteoclastogenesis was unaffected (Fig. S6b). Likewise, there was no genotype-dependent difference observed with respect to serum levels of Rankl or CTX-I, a biomarker of bone resorption (Fig. S6c). Most importantly, however, histology of the knee joints revealed that the severity of articular cartilage degeneration after ACLT was strongly reduced with chondrocyte inactivation of Piezo1 (Fig. 5a, b). Namely, the severe OA development found in the operated knee of all *Piezo1*^*fl*^ controls, as quantified by the OARSI scoring system, was significantly less pronounced in mice with chondrocyte-specific Piezo1 inactivation (Fig. 5c). Similarly, the surgery-induced thinning of the articular cartilage was only observed in *Piezo2*^*Col2a1Cre*^ mice and Cre-negative controls (Fig. 5d). The attenuation of OA development in *Piezo1*^*Col2a1Cre*^ mice was also reflected by a significant reduction in the thickness of the subchondral bone as well as decreased synovitis compared with *Piezo1*^*fl*^ or *Piezo2*^*Col2a1Cre*^ mice (Fig. 5e, f).

**Fig. 6:**
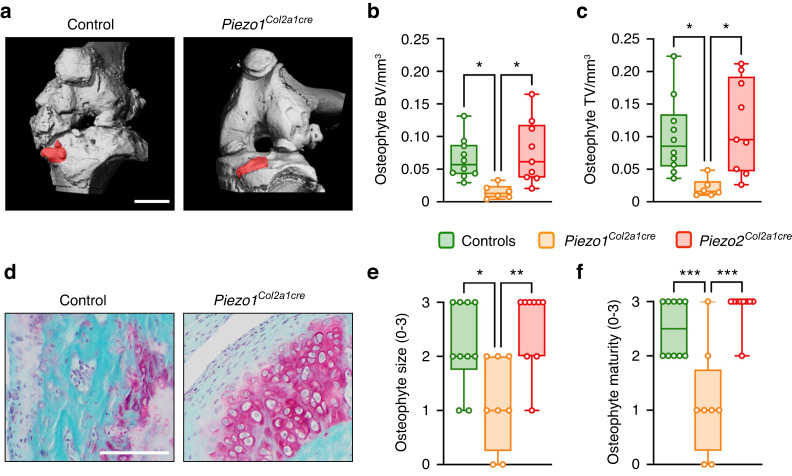
Osteophyte formation and maturation are markedly reduced in Piezo1^Col2a1cre^ mice. **a** Representative µCT images of knee joints after ACLT from control and *Piezo1*^*Col2a1Cre*^ mice showing smaller osteophytes in *Piezo1*^*Col2a1Cre*^ mice, scale bar: 1 mm. **b** µCT-based quantification of the bone volume (BV) of osteophytes and **c** osteophyte total volume (TV). **d** Representative, Safranin-O stained image of tibial osteophytes highlighting the immature cartilaginous osteophyte composition in Piezo1^Col2a1Cre^ mice, scale bar: 100 µm. **e** Quantification of osteophyte size and **f** osteophyte maturity. *n* ≥ 8. Statistical analysis was conducted with one-way ANOVA (Tukey). *P < 0.05, **P < 0.01, ***P < 0.001

Finally, one of the most pronounced effects of chondrocyte-specific Piezo1 inactivation was the prevention of OA-associated periarticular ectopic bone formation, i.e., osteophyte development. While not reduced by chondrocyte-specific inactivation of Piezo2, osteophytes were barely detectable in *Piezo1*^*Col2a1Cre*^ mice (Fig. 6a). In fact, the mean tissue volume and bone volume of osteophytes at the tibia surface of operated knees were significantly reduced by more than 70% specifically in *Piezo1*^*Col2a1Cre*^ mice (Fig. 6b, c). In addition, osteophytes developing in *Piezo1*^*Col2a1Cre*^ mice displayed large areas with persistent chondrocytes (Fig. 6d), which was reflected by a significant reduction in osteophyte size and maturity (Fig. 6e, f). These findings demonstrate that Piezo1 is a major driver of OA progression and that its expression in chondrocytes is a prerequisite for pathological endochondral ossification during osteophyte formation. Given the enormous clinical relevance of understanding OA development in general and osteophyte formation in particular, we next applied immunohistochemistry to localize Piezo1 in intact and diseased human articular cartilage as well as in human osteophyte specimens.

### Expression of Piezo1 in human OA cartilage and osteophytes

**Fig. 7:**
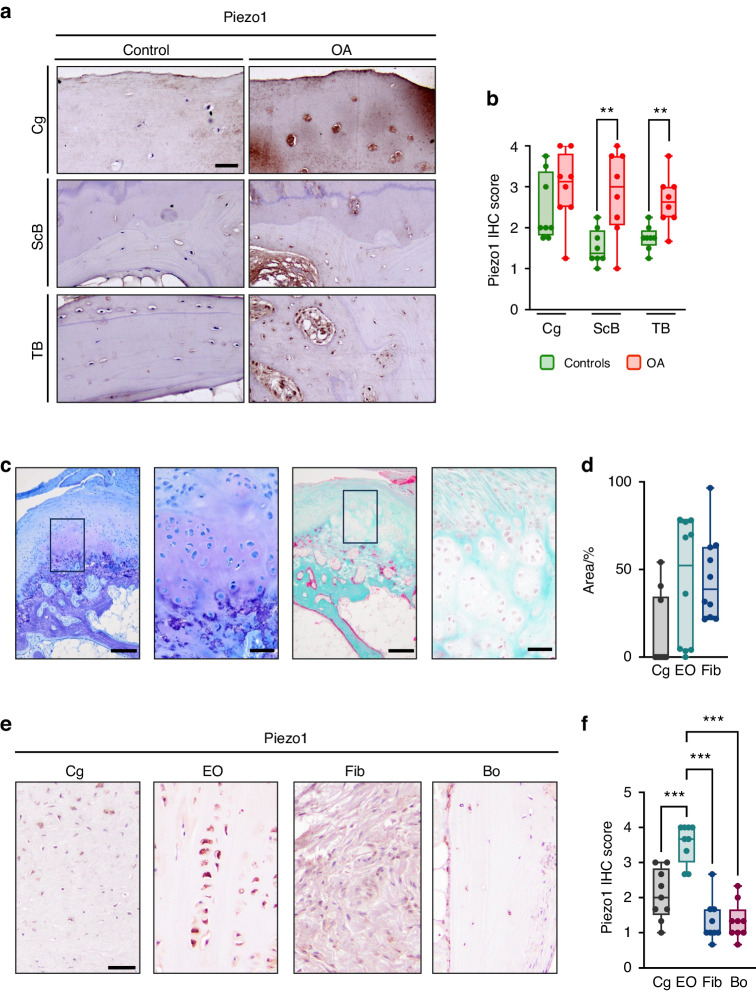
Piezo1 expression is increased in human OA. **a** Representative histological images of femoral head sections stained for Piezo1 by immunohistochemistry, scale bar: 50 µm. **b** Quantification of Piezo1 expression in the articular cartilage (Cg), subchondral bone (ScB), and trabecular bone (TB) (*n* ≥ 8). **c** Representative images of toluidine-blue- (left) and trichrome-goldner-stained (right) sections of human osteophyte specimens. Boxes in the overview images (scale bar: 200 µm) indicate the position of the images shown at higher magnification (scale bar: 50 µm). **d** Histomorphometric quantification of the area of the various regions of the osteophyte rim. Cg: cartilage; EO: zones of endochondral ossification; Fib: fibrotic areas. **e** Representative histological images of human osteophyte sections stained for Piezo1 by immunohistochemistry, scale bar: 50 µm. **f** Quantification of Piezo1 expression in cartilage (Cg), zones of endochondral ossification (EO), fibrotic areas (Fib) and bone (Bo) (*n* ≥ 8). Statistical analysis was conducted with Student’s *t* test comparing control and OA samples (**b**), or one-way ANOVA (Tukey) (**f**). **P < 0.01, ***P < 0.001

To analyze the protein expression of human Piezo1, we took advantage of histological sections obtained from patients with OA and of control specimens (Fig. S7). We found that Piezo1 was more abundant in the subchondral and trabecular bone regions of OA sections, whereas its expression in articular chondrocytes was comparable to control sections (Fig. 7a, b). Since in addition to articular cartilage degeneration and subchondral bone plate alterations, osteophytes contribute to the clinical manifestation of OA, we analyzed human osteophytes from the femoral head and neck region affected by OA. Our analysis revealed distinct regions within osteophytes, wherein we observed fibrotic and cartilaginous components along with evidence of endochondral ossification (Fig. 7c, d). Given the known involvement of Piezo1 in endochondral ossification, we assessed the expression of Piezo1 in various osteophyte regions (Fig. 7e). Remarkably, we detected an elevated abundance of Piezo1 particularly in cartilage and endochondral ossification zones (Fig. 7f). Collectively, these results suggest that Piezo1 stimulation promotes articular cartilage degeneration as well as osteophyte development in humans, and that antagonism of Piezo1 or its downstream effectors could be a molecular approach to limit these pathologies.

### Piezo1 activation causes a specific transcriptional response in chondrocytes

**Fig. 8:**
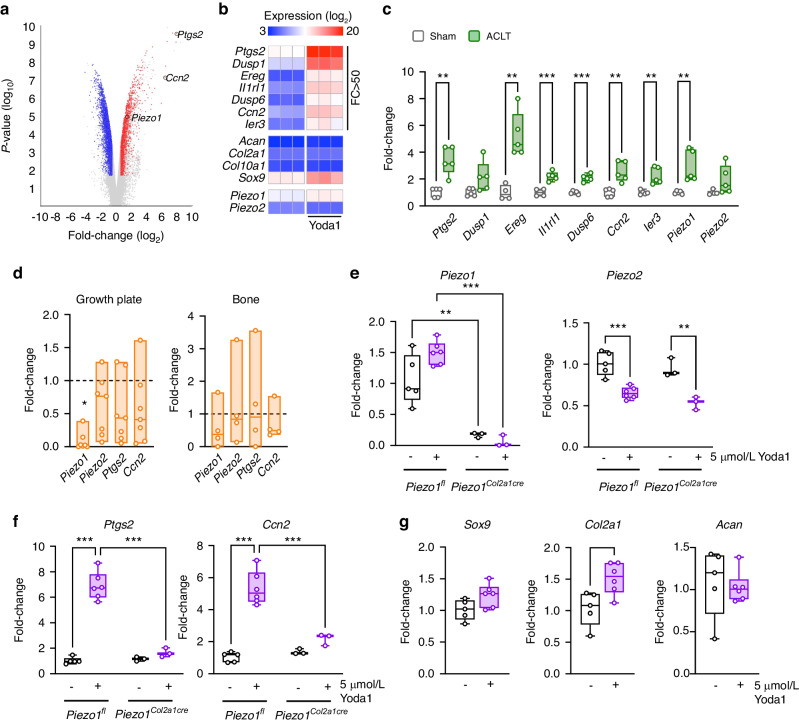
Piezo1 activation results in a specific transcriptional response. **a** Volcano plot showing differentially expressed genes in chondrogenic ATCD5 cells treated with the Piezo1 agonist Yoda1 (5 μM) or DMSO for 6 h. **b** Heat map showing the seven genes with a more than 50-fold change (FC > 50) after Yoda1 treatment, four established chondrocyte markers, as well as *Piezo1* and *Piezo2*. Data are shown as log_2_ expression values. **c** Expression analysis by qRT-PCR for the indicated genes in knee joints of mice 8 weeks after ACLT compared to sham-operated controls (*n* = 5). **d** Expression analysis by qRT-PCR of the indicated genes in growth plate (left) and bone (right) of *Piezo1*^*Col2a1Cre*^ mice relative to controls (indicated by dashed line). **e** Expression analysis by qRT-PCR of *Piezo1* and *Piezo2* in cultured primary chondrocytes isolated from *Piezo1*^*fl*^ and *Piezo1*^*Col2a1Cre*^ mice treated with Yoda1 or DMSO. **f** Expression analysis by qRT-PCR of *Ptgs2* and *Ccn2* in the same samples. **g** Expression analysis of *Sox9*, *Col2a1* and *Acan* in cultured primary chondrocytes from control mice treated with Yoda1 or DMSO. Statistical analysis was conducted with Student’s *t* test (**c**, **g**) or two-way ANOVA (Tukey) (**e**, **f**). *P < 0.05, **P < 0.01, ***P < 0.001

Whereas the skeletal phenotypes in *Piezo1*^*Col2a1Cre*^ mice were remarkably evident, the molecular mechanisms explaining the observed differences towards *Piezo1*^*fl*^ controls remain elusive. Therefore, to screen for potentially relevant Piezo1 downstream targets in chondrocytes we performed genome-wide expression analysis using chondrogenic ATDC5 cells that were treated for 6 h with the Piezo1 agonist Yoda1 (Fig. 8a). We hereby observed a specific transcriptional response with 7 genes displaying a more than 50-fold induction after Yoda1 treatment, whereas other chondrocyte markers were not regulated to a similar extent (Fig. 8b). Since *Ptgs2*, encoding prostaglandin-endoperoxide synthase 2, as well as *Ccn2*, encoding connective tissue growth factor (Ctgf), have previously been implicated in regulating chondrogenic functions,^34–36^ we subsequently confirmed the regulation of both genes by qRT-PCR. Here we additionally observed that the Yoda1-mediated induction of *Ptgs2* and *Ccn2* was dose-dependently inhibited by the Yap/Taz signaling inhibitor Verteporfin (Fig. S8a). In contrast, the comparably moderate 2-fold induction of *Sox9*, encoding a key transcription factor promoting chondrogenesis,^37^ that was observed after short-term Yoda1 administration, was not reduced by the presence of Verteporfin. Since a pathogenic role of Ccn2/Ctgf has previously been suggested in the context of OA progression,^38,39^ and as another differentially expressed gene (*Ereg*, encoding epiregulin) was recently identified as a potential OA modifier in an unbiased transcriptomic profiling approach,^40^ we further determined gene expression in articular cartilage from wildtype mice after ACLT surgery. More specifically, when we applied qRT-PCR to monitor the expression of the seven genes displaying the strongest induction after Yoda1 treatment of ATDC5 cells, five of these were more strongly expressed in OA knee joints (Fig. 8c). A similar difference was also observed for the expression of *Piezo1*, but not of *Piezo2*. These data identified *Ccn2* and other potentially OA-related genes as transcriptional targets of Piezo1-activated signaling in chondrocytes, which we additionally verified using primary cell cultures.

Consistent with the significantly reduced expression of *Piezo1* in the growth plates of *Piezo1*^*Col2a1Cre*^ mice (Fig. 8d), we found by qRT-PCR expression analysis of cultured primary chondrocytes that cells from *Piezo1*^*Col2a1Cre*^ mice barely express *Piezo1*, whereas *Piezo2* expression was not significantly changed compared to *Piezo1*^*fl*^ chondrocytes (Fig. 8e). In contrast to *Piezo1*, *Piezo2* expression was moderately, yet significantly reduced by short-term administration of Yoda1. Most importantly however, by comparing the response of primary chondrocytes from *Piezo1*^*fl*^ and *Piezo1*^*Col2a1Cre*^ towards Yoda1, we were able to confirm that the more than 5-fold induction of *Ptgs2* and *Ccn2* in these cells was Piezo1-dependent, as the Yoda1 response was abolished in *Piezo1*^*Col2a1Cre*^ primary chondrocytes (Fig. 8f). A significant impairment of Yoda1-induced gene expression in *Piezo1*^*Col2a1Cre*^ cells was also found for the other above-described genes, with the prominent exception of *Ereg*, which appears to be regulated in a Piezo1-independent manner (Fig. S8b). It is also worthwhile to state that *Sox9* expression was not significantly affected by Yoda1 treatment of primary chondrocytes, similar to *Acan*, whereas *Col2a1* expression was slightly, but significantly increased (Fig. 8g).

While we have previously identified *Ptgs2* as a Piezo1-regulated gene in the osteoblast cell line MC3T3-E1^25^, the Piezo1-dependent induction of *Ccn2* expression in chondrocytes was not only unexpected, but could also be a functional relevance, as there a numerous functions described for Ccn2/Ctgf in the context of chondrogenesis.^35^ In the present study, we took advantage of an established transdifferentiation assay, again using ATDC5 cells, where after 7 days of culturing in chondrogenic medium, the culture conditions are switched to promote osteogenic differentiation.^41^ Here we found that the addition of Ccn2/Ctgf significantly induced the expression of osteogenic genes and markers (*Runx2*, *Sp7*, *Col1a1* and *Alpl*), but also of chondrogenic markers (*Acan*, *Col2a1*) (Fig. S9a–c). Although our collective findings identified Ccn2/Ctgf as a candidate downstream mediator of Piezo1 in chondrocytes, their physiological relevance remains to be confirmed in vivo, for instance by generating additional mouse models with combined genetic modifications.

**Fig. 9:**
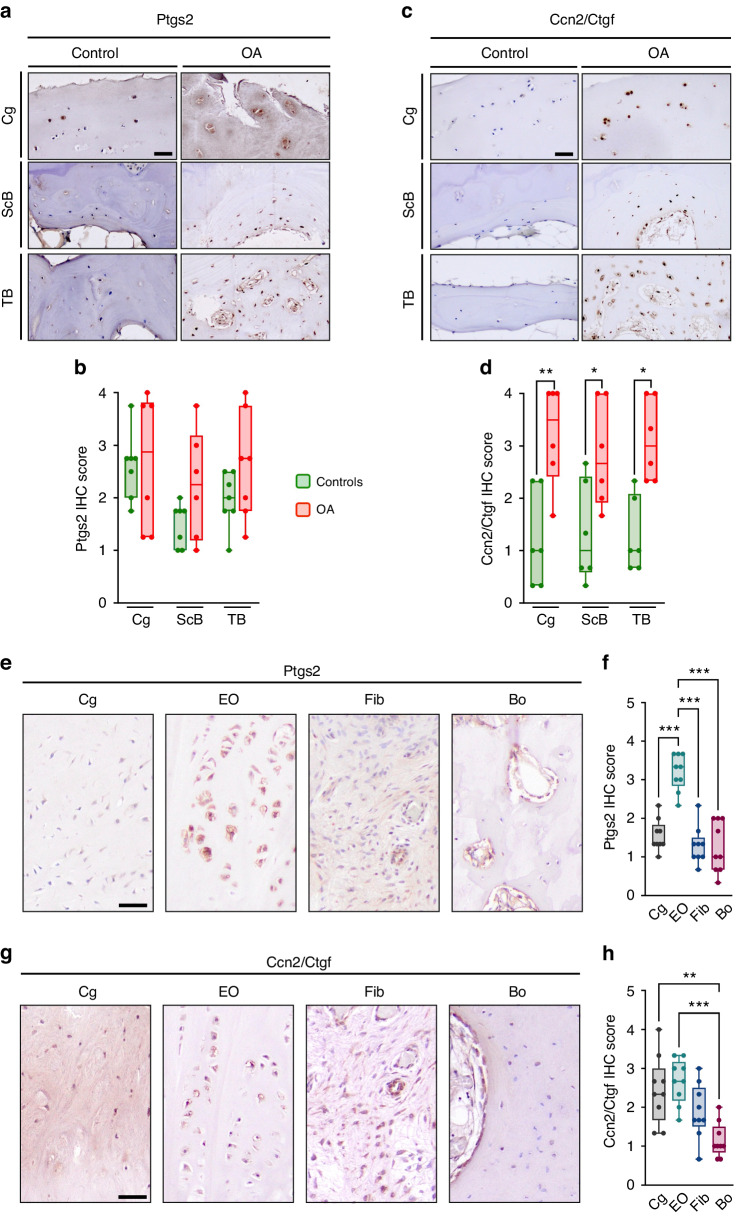
Ptgs2 and Ccn2/Ctgf are expressed in human osteophytes. **a** Representative histological images of femoral head sections stained for Ptgs2 by immunohistochemistry, scale bar: 50 µm. **b** Quantification of Ptgs2 expression in the articular cartilage, subchondral bone, and trabecular bone. **c** Representative histological images of femoral head sections stained for Ccn2/Ctgf by immunohistochemistry, scale bar: 50 µm. **d** Quantification of Ccn2/Ctgf expression in the articular cartilage, subchondral bone, and trabecular bone (*n* ≥ 8). **e** Representative histological images of human osteophyte sections stained for Ptgs2 by immunohistochemistry, scale bar: 50 µm. **f** Quantification of Ptgs2 expression in cartilage (Cg), zones of endochondral ossification (EO), fibrotic areas (Fib) and bone (Bo). **g** Representative histological images of human osteophyte sections stained for Ccn2/Ctgf by immunohistochemistry, scale bar: 50 µm. **h** Quantification of Ccn2/Ctgf expression in cartilage (Cg), zones of endochondral ossification (EO), fibrotic areas (Fib) and bone (Bo) (*n* ≥ 8). Statistical analysis was conducted with Student’s *t* test comparing control and OA samples (**b**, **d**), or with one-way ANOVA (Tukey) (**f**, **h**), *P < 0.05, **P < 0.01, ***P < 0.001

In the present manuscript, however, we took advantage of the human specimens described above to address the question if and where either Ptgs2 or Ccn2/Ctgf are expressed in control and OA sections. With respect to Ptgs2, we found no significant differences between control and OA sections in any of the joint compartments (Fig. 9a, b). In contrast, Ccn2/Ctgf was significantly overproduced in articular chondrocytes as well as in subchondral and trabecular osteoblasts/osteocytes from patients with OA (Fig. 9c, d). In sections from osteophytes, the differences between the different regions were more pronounced for Ptgs2, which displayed a significant induction in the endochondral ossification regions compared to other sites (Fig. 9e, f). However, the expression of Ccn2/Ctgf was also significantly higher in cartilage and endochondral ossification regions, when compared to fibrotic areas or bone (Fig. 9g, h). Since Piezo1, as described above, displayed a similar distribution in osteophyte sections, our collective data strongly suggest that Piezo1 expression in chondrogenic cell types promotes osteogenic osteophyte maturation, which is potentially mediated by the induction of specific genes, such as *Ptgs2* or *Ccn2*. Finally, since osteophyte development can be regarded as a pathological endochondral ossification event, it is likely that the same mechanisms that are physiologically controlled by *Piezo1* in chondrocytes, as evidenced for vertebral bodies and ribs, are also utilized during pathologic osteophyte maturation.

## Discussion

Our observations provide novel insights into the role of Piezo1 in chondrocytes and allow several conclusions with high relevance for basic and clinical research. First, we demonstrate that Piezo1, unlike Piezo2, has a unique function in chondrocytes, where its deficiency only moderately affects skeletal growth, but strongly impairs postnatal trabecular bone formation. Second, we show that chondrocyte expression of Piezo1 is not only physiologically required for endochondral ossification, but also for pathological processes linked to OA progression and osteophyte formation. Third, our genome-wide expression analysis using a chondrogenic cell line identified specific Piezo1-regulated genes, among which *Ccn2* has been suggested to be involved in OA progression.^38,39^ Finally, the increased expression of Piezo1 and Ccn2/Ctgf detected in articular cartilage, subchondral bone, and osteophytes from OA patients underscores the clinical relevance of our findings obtained in mice.

Our present study is based on previous findings obtained in mice lacking Piezo1 and/or Piezo2 in different skeletal cell types.^25^ Most importantly, since we identified a unique skeletal phenotype in the *Piezo1*^*Runx2Cre*^ model, where we observed a lack of trabecular bone below the chondrogenic growth plates, unlike it was the case in *Piezo1*^*Dmp1Cre*^ animals, we additionally took advantage of the *Col2a1*^*Cre*^ transgenic mouse line to generate *Piezo1*^*Col2a1Cre*^ mice. In a first set of 12-week-old animals we thereby confirmed that chondrocyte-specific inactivation of Piezo1 impairs trabecular bone formation and we also identified a high incidence of rib fractures in 2-week-old *Piezo1*^*Col2a1Cre*^ mice, based on contact radiographs.^25^ This led us to deepen our studies in order to define the onset of the observed phenotypes, to rule out a potential role of Piezo2, to histologically analyze the cellular disturbances also in the rib bones, and to analyze older *Piezo1*^*Col2a1Cre*^ mice for articular cartilage pathologies.

We thereby demonstrated that Piezo1, unlike Piezo2, has a specific function in chondrocytes. In fact, whereas Piezo1 is not required for the proper alignment of the different chondrocyte populations in the growth plate, it is essential for trabecular bone formation in the process of endochondral ossification. The *Piezo1*^*Col2a1Cre*^ phenotype of reduced trabeculae below the growth plate develops postnatally, and since we did not identify an increased number of TRAP-positive cells in these areas, we hypothesize that Piezo1 inactivation in chondrocytes impairs their transdifferentiation into bone-forming osteoblasts.^18^ In the present study we also provide a more detailed examination of the *Piezo1*^*Col2a1Cre*^ rib bones, in which histological analysis, together with µCT-based quantification, identified a similar reduction of trabecular bone formation as it was found in the spine. Importantly, whereas rib fractures were absent in most *Piezo1*^*Col2a1Cre*^ mice immediately after birth, all 7-day-old animals displayed multiple rib fractures, generally located close to the growth plates. The presence of a fracture callus at that age, together with the steadily decreasing number of fractured ribs with increasing age, demonstrated that due to the effective fracture healing this major phenotype is transient. Therefore, also explained by the fact that Piezo1 deficiency did not affect the cortical compartment of the rib bones, there were no fractures observed in adult *Piezo1*^*Col2a1Cre*^ mice, which enabled us to study articular cartilage with respect to OA-like pathologies. Here we observed that Piezo1 inactivation in chondrocytes significantly reduces OA severity and osteophyte formation, either in unchallenged 60-week-old mice or in 20-week-old animals following ACLT.

Although OA represents one of the most prevalent disorders in the aging population, there is still no specific molecular treatment available to decelerate OA progression.^42^ Therefore, after experiencing chronic pain and loss of joint mobility for an extended period of time, the vast majority of patients require joint replacement surgery. This explains the necessity to provide a better molecular understanding of OA, either by large-scale genetic studies in humans or by assessing OA development in genetically modified mouse models. In the latter, OA is typically induced by surgery to destabilize the knee joint. This can be achieved either by destabilization of the medial meniscus (DMM) or by ACLT as applied in the present study. At this point it is again important to refer to the two recently published studies, where DMM was applied to assess the involvement of Piezo proteins in OA progression.^31,32^ In the first study, the authors did not observe a difference in OARSI scores between *Piezo1/2*^*Gdf5Cre*^ mice and *Piezo1/2*^*fl*^ controls, which apparently contradicts our findings.^31^ In the second study, however, OA development was significantly attenuated in mice with selective Piezo1 inactivation, which was achieved with an inducible system using the *Acan*^*CreERT2*^ transgenic mouse line.^32^ At present, we can only speculate about the reason for the divergent results reported in the two studies, yet the most obvious difference is related to the distinct *Cre* lines that were used to inactivate Piezo1. Therefore, given the opposing results published to date, it is even more important that our study, albeit being performed with another *Cre* line, confirms the previously suggested detrimental role of Piezo1 for OA development in a similar, but slightly different experimental setting. Likewise, a rare missense variant in *PIEZO1* was recently identified in an extensive genome-wide association approach to be negatively correlated with hip OA in humans.^43^ Therefore, our data obtained in mice, showing that chondrocyte inactivation of Piezo1, but not of Piezo2, attenuates OA progression and osteophyte formation, provide additional genetic evidence to demonstrate the relevance of one of the two known Piezo proteins for articular cartilage integrity.

In the context of the Piezo1-regulated genes, it is further relevant to state that surgical OA induction has already been performed in mice lacking Ptgs2 or Ccn2/Ctgf. Whereas Ptgs2 deficiency did not influence OA progression, postnatally induced Ccn2 deficiency attenuated articular cartilage degeneration.^39,44^ Together with our immunohistochemistry results obtained from human OA specimens, these data suggest that Ccn2/Ctgf, in contrast to Ptgs2, represents a major driver of articular cartilage degeneration and subchondral bone alterations. However, our analysis of osteophytes revealed a notable Ptgs2 expression, particularly in zones of endochondral ossification. Thus, Ccn2/Ctgf may be involved in the modifications of cartilage, subchondral bone, and the process of osteophyte formation and maturation, whereas Ptgs2, also known as cyclooxygenase-2 (Cox-2), may be primarily involved only in the process of osteophyte formation and maturation. Since a humanized monoclonal antibody against Ccn2/Ctgf (Pamrevlumab) has already been approved for the treatment of idiopathic pulmonary fibrosis, it appears reasonable to assess its applicability for the treatment of OA.^45,46^ Additionally, Cox-2 inhibitors may be evaluated as potential inhibitors of osteophyte formation.

Given the remarkable phenotypic differences that we observed between *Piezo1*^*Col2a1Cre*^ and *Piezo2*^*Col2a1Cre*^ mice, it can also be concluded that only Piezo1 plays a major role in chondrocytes, which also applies to the physiologically relevant postnatal formation of trabecular bone below the growth plate. Based on a recently published study, however, it is likely that the expression of Piezo2 in sensory neurons plays a key role in nociception, which is also clinically relevant in the context of OA-associated pain.^47^ With respect to skeletal cell types, a Piezo1-specific function has previously been demonstrated for osteocytes, since *Piezo2*^*Dmp1Cre*^ mice, unlike *Piezo1*^*Dmp1Cre*^ mice, did not display a low bone mass phenotype.^22,25^ From a pharmacological perspective, this specificity should not be underestimated, especially since more selective inhibitors than GsMTx4 can potentially be developed. Nonetheless, it has to be taken into consideration that Piezo1 does not only control pathological processes, but that it is also required in various cell types outside the skeleton to control key functions in the organism.^4^ Therefore, functional antagonism of Piezo1 likely requires intraarticular injection, in order to avoid detrimental side effects as previously performed in mice with GsMTx4.^30^ Moreover, since Piezo1 activation has recently been shown to promote pathological bone formation in ankylosing spondylitis, it is conceivable to speculate that similar therapeutic strategies are applicable to additional skeletal pathologies.^48^

Despite the obviously attenuated OA development in *Piezo1*^*Col2a1Cre*^ mice reported here, our study has several limitations. First, we can only speculate about the nature of the mechanical triggers that activate Piezo1 in physiological and pathological endochondral ossification processes. Whether alterations in matrix stiffness and/or joint instability are the primary drivers needs to be further investigated. Second, further proof is needed to show that the near absence of postnatal trabecular bone formation in *Piezo1*^*Col2a1Cre*^ mice is indeed the consequence of impaired chondrocyte transdifferentiation into bone-forming osteoblasts. To address this question, lineage tracing experiments are required, where an inducible chondrocyte deficiency of *Piezo1* is combined with a Cre-activated reporter gene.^49^ Third, it remains to be determined, if Ccn2/Ctgf is indeed a relevant physiological and pathological downstream effector of Piezo1. Ideally, this causality could be shown by modulation of the *Piezo1*^*Col2a1Cre*^ phenotypes through transgenic overexpression or inactivation of *Ccn2* or by taking advantage of an established mouse model allowing the modulation of *Ccn2* expression levels.^50^ Finally, we did not show that pharmacologic blockade of Piezo1 attenuates OA in our own experimental setting. However, given the previously published study showing a protective effect by intraarticular injection of GsMTx4,^30^ we truly believe, also in terms of animal welfare concerns, that it is not required to independently reproduce these data.

In summary, our results demonstrate a strong and unique impact of chondrocyte-specific Piezo1 deficiency in mice, which provides the basis for additional studies related to a molecular understanding of OA, in mice and humans. We show that Piezo1 expression in chondrocytes is a prerequisite for trabecular bone formation in the context of endochondral ossification. Moreover, our data indicate that the same function of Piezo1 promotes osteophyte formation, which develop by pathological endochondral ossification. Although extensive efforts may still be required to ultimately establish a specific molecular treatment limiting OA development and progression, it appears that antagonism of Piezo1 might be a promising therapeutic approach in this regard.

## Materials and methods

### Mice

Generation and genotyping of *Piezo1*^*fl/fl*^ and *Piezo2*^*fl/fl*^ mice have been described previously.^10,51^ *Col2a1Cre* mice^52^ were purchased from the Jackson Laboratory (Bar Harbor, ME, USA; #003554). We crossed *Piezo1*^*fl/fl*^, *Piezo2*^*fl/fl*^ or both with *Col2a1Cre* mice and analyzed animals from the resulting mouse strain (STOCK *Piezo1*^tm1c(KOMP)Wtsi^ *Piezo2*^tm2.2Apat^ Tg(Col2a1-cre)1Bhr/Uke). To exclude a possible influence of genetic background, all analyses were performed with the corresponding Cre-negative littermates. Mice were housed in a specific pathogen-free environment with a 12 h light-dark cycle, 45% to 65% relative humidity, and 20 °C to 24 °C ambient temperature in open or individually ventilated cages with wood chip bedding and nesting material in groups of no more than six animals. Mice had ad libitum access to tap water and standard rodent food (Altromin Spezialfutter GmbH & Co. KG, Lage, Germany;1328 P). Animal breeding and all experimental procedures with defined humane endpoints were performed with approval from the animal care committees of the University Medical Center Hamburg-Eppendorf and the Hamburg Ministry of Justice and Consumer Protection (N033/2022, N031/2021). No preregistration was performed.

### Anterior cruciate ligament transection (ACLT)

The anterior cruciate ligament transection (ACLT) procedure was performed on the right knee joint of 12 weeks old female *Piezo1*^*Col2a1cre*^ (*n* = 8) and *Piezo2*^*Col2a1cre*^ (*n* = 9) mice as well as Cre-negative littermates (*n* = 10) to induce OA, as previously described.^53^ To minimize confounders, batch surgeries were performed with the inclusion of different genotypes per batch. Briefly, mice were anesthetized using isoflurane (4% isoflurane for induction and 1%–2% for maintenance), and the right knee joint was accessed via a medial parapatellar incision. Using a scalpel with a pointed blade, the anterior cruciate ligament of the knee joint was transected under vision using 2.5x magnification glasses. Complete transection was confirmed by a positive anterior drawer test, indicating successful ACLT. Prior to surgery, mice were administered a prophylactic dose of 150 mg/kg clindamycin subcutaneously to reduce the risk of infection. To manage post-operative pain, mice were given 0.1 mg/kg buprenorphine subcutaneously. Both treatments were administered in a sterile manner to minimize the risk of infection and contamination. Additionally, 2 g/L metamizole was added to the drinking water for three days pre- and post-surgery. The contralateral (left) knee joints served as a non-operated control to account for any systemic changes or individual differences that may affect the study outcomes. Non-operated control joints were not subjected to any surgical intervention and were processed identically to ACLT joints. After surgery, the welfare of mice was assessed daily based on overall appearance and body weight. One animal was excluded from the analysis due to a post-surgery complication. Animals were sacrificed 8 weeks post-surgery via CO_2_ intoxication. After dissection, knee joints were aligned at an angle of 90° and skeletons were fixed in 3.7% PBS buffered formalin for 48 h. Contact radiography was performed for all animals after fixation. All endpoint measurements described below were performed in a blinded fashion.

### Micro-computed tomography (µCT)

To determine the bone microstructure in knee joints, micro-computed tomography (µCT) was performed using a µCT 40 cone-beam system (SCANCO Medical AG, Brüttisellen, Switzerland) with a voxel size of 10 µm. The X-ray tube was operated at 55 kVp and 145 µA intensity. To investigate OA-related changes in both the ACLT and non-operated control knees, the subchondral bone and osteophytes of the tibia were evaluated using µCT. Trabecular bone microarchitecture parameters were analyzed in the subchondral region extending until the growth plate. For determination of osteophyte parameters manual segmentation was applied.

Due to frequent rib fractures in 7-day-old mice, rib morphology was not assessable by standard µCT. To determine reasons for early fracturing, the trabecular and bone microstructure of ribs in newborn (P0) and 6-week-old *Piezo1*^*Col2a1Cre*^ mice was analyzed. Ribs were scanned using a desktop µCT system (Skyscan 1272, Bruker, Kontich, Belgium) at a resolution of 1.4 µm and 3.4 µm voxel size for P0 and 6-week-old mice, respectively. Per sample, the values obtained from four ribs were averaged. The X-ray tube was operated at a voltage of 50 kVp and an intensity of 200 µA. For quantification, the trabecular and cortical microstructure was evaluated separately in a region of interest from 0.5 mm to 8 mm distal (Fig. 2d) of the growth plate. Specifically, the cortical thickness (Ct.Th in µm) and the bone volume fraction (BV/TV in %) were determined.

### Undecalcified histology and histomorphometry

Bone samples were dehydrated in increasing concentrations of ethanol and embedded in methylmethacrylate for undecalcified histology, using previously described protocols.^25^ For knee joints, 4 µm thick coronal plane sections, were obtained and stained using Safranin-O, trichrome Goldner, toluidine blue, hematoxylin-eosin (HE), and Movat Pentachrome staining as described before.^54–56^ The severity of OA-related changes in all four compartments of the knee joint was assessed using the Osteoarthritis Research Society International (OARSI) scoring system for mice, with scores ranging from 0 (normal joint) to 24 (severe OA).^57^ Each compartment of the joint was evaluated separately and then summed to obtain an overall score for each mouse. The assessment of the osteochondral unit included cartilage thickness, calcified cartilage thickness and subchondral bone plate thickness, which were assessed for each compartment of the joint separately. For each parameter, the values of the compartments were averaged to provide a single value per knee joint. The histopathological synovitis score was used to assess inflammatory changes in the knee joints.^58^ Briefly, the enlargement of synovial lining cells and their density were evaluated individually. The formation of osteophytes was evaluated using a scoring system based on osteophyte size and maturity, as previously described.^59^ For histomorphometric analysis of knees and lumbar spine, sagittal plane sections were obtained and stained with toluidine blue, Safranin-O, and the von Kossa/van Giesson procedure as described previously.^60^ Trabecular parameters (Bone volume fraction (BV/TV in %), trabecular number (Tb.N in mm^−1^)) were quantified using the BioQuant Osteo Software (BIOQUANT Image Analysis Corp., Nashville, TN, USA) in accordance with the ASBMR guidelines.^61^ Cellular histomorphometric parameters [Number of osteoblasts per bone perimeter (N.Ob/B.Pm in mm^−1^), number of osteoclasts per bone perimeter (N.Oc/B.Pm in mm^−1^), osteoblast surface per bone surface (Ob.S/B.S in %) and osteoclast surface per bone surface (Oc.S/B.S in %)] were quantified in undecalcified toluidine blue stained sections of knees after ACLT surgery and lumbar spine of 6-week-old mice using the Osteomeasure system (OsteoMetrics, Inc., Atlanta, GA, USA) in accordance with the ASBMR guidelines.^61^ Growth plate thickness and chondrocyte number were measured on toluidine-stained sections using the Osteomeasure system (OsteoMetrics).

### Serum levels of bone resorption markers

The levels of bone resorption markers in the serum of ACLT mice after final exsanguination were measured using the respective enzyme-linked immunosorbent assays (ELISA) according to the manufacturer’s instructions. Levels of receptor activator of nuclear factor kappa-Β ligand (Rankl) were measured using the respective Quantakine Elisa Kit (R&D Systems, Minneapolis, MN, USA; MTR00). The concentrations of C-terminal telopeptide of type I collagen (CTX-I) were determined using the RatLaps kit (Immunodiagnostic Systems Holdings Ltd., Boldon, UK; AC-06F1).

### Cell culture

Murine chondrogenic ATDC5 cells (Sigma-Aldrich; 99072806) were cultured in DMEM:F12 (Gibco, Billings, MT, USA; 11320-033) with 5% FCS (Gibco; 10437-028) and 100 U/mL penicillin and streptomycin (Gibco; 15140122) at 37 °C, 5% CO2 and 95% relative humidity.

Primary murine chondrocytes were isolated from rib cage cartilage of 10-day-old mice by collagenase (Sigma-Aldrich; C9891) digestion in DMEM:F12 and cultured in DMEM:F12 with 10% FCS and 50 μg/mL ascorbic acid at 37 °C, 5% CO_2_ 5% O_2_ and 95% relative humidity.

### Treatment with Yoda1 and Verteporfin

ATDC5 cells were seeded at a density of 4.3 × 10^4^/cm^2^. Primary murine chondrocytes were seeded at a density of 6.3 × 10^4^/cm^2^ and cultured for four days until confluent. Cells were serum-starved overnight and subsequently stimulated for 6 h in serum-free growth medium with the Piezo1 agonist Yoda1 (Tocris Bioscience, Bristol, UK; 5586, 5 μmol/L) and/or the YAP inhibitor Verteporfin (Selleck Chemicals LLC, Houston TX; S1786). Control cells were treated with an equal amount of DMSO.

### In vitro RNA expression and transcriptome analysis

RNA from cultured cells was isolated and subjected to DNase digestion using the NucleoSpin RNA kit (Macherey-Nagel GmbH & Co. KG, Düren, Germany) according to the manufacturer’s instructions. Concentration and quality of RNA were measured using a NanoDrop ND-1000 system (Thermo Fisher Scientific, Waltham, MA, USA) and the TapeStation 2200 system (Agilent Technologies, Santa Clara, CA, USA). Genome-wide expression analysis was performed using the Clariom D system (Thermo Fisher Scientific) as described previously.^25^ In brief, the Clariom D assay system (Thermo Fisher Scientific) was used with 100 ng of total RNA as starting material to generate 2nd-cycle single-stranded complementary DNA (ss-cDNA). 5.5 μg of ss-cDNA were used for gene chip hybridization (Clariom D, mouse, Thermo Fisher Scientific). Gene chips were washed and stained using the Affymetrix Fluidics Station 450 and scanned with the Affymetrix Gene Chip Scanner 7 G (both Affymetrix, Santa Clara, CA, USA). Data were analyzed in the Transcriptome Analysis Console software (TAC 4.0; Thermo Fisher Scientific) using default analysis settings (version1) and Gene + Exon-signal space transformation-robust multiarray analysis (SST-RMA) as summarization. Full gene expression datasets have been deposited at the GEO database (National Center for Biotechnology Information [NCBI], Bethesda, MD, USA; https://www.ncbi.nlm.nih.gov/geo/) with accession number GSE230071.

For qRT-PCR expression analysis, 500 ng of total RNA was reverse transcribed using Verso cDNA Synthesis Kit (Thermo Fisher Scientific) according to the manufacturer’s instructions using oligo-dT primers. Quantitative expression analysis was performed using a StepOnePlus system and predesigned TaqMan gene expression assays (Thermo Fisher Scientific). *Gapdh* expression was used as the reference housekeeping gene. The following assays were used: *Ptgs2* (Mm00478374_m1), *Ccn2* (Mm01192933_g1), *Sox9* (Mm00448840_m1), *Dusp1* (Mm00457274_g1), *Ereg* (Mm00514794_m1), *Il1rl1* (Mm00516117_m1), *Dusp6* (Mm00518185_m1), *Ier3* (Mm00519290_g1), *Piezo1* (Mm01241549_m1), *Piezo2* (Mm01265861_m1), *Col2a1* (Mm00491889_m1), *Acan* (Mm00545794_m1). Data analysis was performed according to the delta-delta comparative threshold cycle (2^-ΔΔCT^) method, and results are shown as fold-change expression values relative to controls.

### Transdifferentiation assay with Ccn2/Ctgf

The transdifferentiation assay was performed as described before.^41^ Briefly, ATDC5 cells were cultured in chondrogenic differentiation medium for 7 days and thereafter cultured in osteogenic differentiation medium containing 50 ng/mL recombinant human Ctgf (PeproTech Inc., Rocky Hill, NJ, USA). Cells were harvested after 24 and 48 h of osteogenic differentiation, respectively. RNA from cell lysates was isolated using the RNeasy Mini Kit and cDNA was generated using Omniscript Reverse Transcriptase (both Qiagen). qRT-PCR expression analysis was performed using Platinum™ SYBR™ Green qPCR SuperMix-UDG and ROX™ Reference Dye (both Thermo Fisher Scientific). The primer sequences of the analyzed genes are shown in Table S1. *B2m* expression was used as the reference housekeeping gene. Data analysis was performed according to the delta-delta comparative threshold cycle (2^-ΔΔCT^) method, and results are shown as fold-change expression values relative to controls.

### In vivo RNA expression analysis

For expression analysis after OA induction, twelve-week-old C57BL/6 J wildtype mice were subjected to ACLT as described above, or a sham operation in which only the medial parapatellar incision was performed. Whole knees were carefully isolated from 20-weeks-old C57BL/6 J mice at 8 weeks after ACLT or sham surgery. Tissue was snap frozen in liquid nitrogen, crushed with mortar and pestle followed by homogenization in TriFast (Peqlab, Erlangen, Germany; 30–2010) using an UltraTurrax T25 (IKA, Staufen, Germany). RNA isolation, cDNA synthesis and qRT-PCR were performed as described above for cultured cells.

For expression analysis of growth plate and bone samples, tibia and femur were isolated from 12-day-old mice. Distal femoral and proximal tibial growth plates and cortical bone samples were isolated using ophthalmic scalpels under a stereo microscope. Tissue was snap-frozen in liquid nitrogen and homogenized using a micropestle. RNA was isolated using a NucleoSpin RNA XS kit (Macherey Nagel GmbH & Co. KG; 740990.50). CDNA synthesis and qRT-PCR were performed as described above.

### Human specimens

Eight femoral heads were collected from patients with primary OA undergoing total hip arthroplasty, comprising of 4 women and 4 men. Control specimens were also obtained from eight individuals (5 women and 3 men) during autopsy. Osteophytes were identified and excised from the margins of the femoral head specimens. The identification of osteophytes was done macroscopically and based on the characteristic morphological features of osteophytes, including their firmness and their localized growth at the joint margins. An excision margin of 1 mm was maintained around the osteophytes to ensure that articular cartilage and normal bone were not included in the excised material. All samples were fixed in 3.7% formaldehyde within 24 h of death or resection. To prepare histological sections, the samples were cut along the coronal plane through the fovea capitis femoris using a diamond-coated saw, with the medial part of the femoral head selected for analysis. Both decalcified and undecalcified histology specimens were prepared. For undecalcified histology, the specimens were embedded in methylmethacrylate, cut into 4 µm sections, and stained with toluidine blue, von Kossa, and Safranin-O. The fractions of the different components of the osteophyte, specifically the osteophyte rim covered with cartilage, zones of endochondral ossification, and fibrotic zones, were manually traced and quantified using the Osteomeasure system (OsteoMetrics, Inc., Atlanta, GA, USA).

### Immunohistochemistry

For immunohistochemistry of human samples, femoral head and osteophyte specimens were prepared as described above and were decalcified in an EDTA-based solution (Usedecalc, Medite, Orlando, USA) for 2 weeks at 20 °C. Decalcified samples were dehydrated in increasing concentrations of ethanol and embedded in paraffin. Coronal sections (4 μm) were prepared from the decalcified specimens. Heat-mediated antigen retrieval was performed at 60 °C in a water bath overnight. Endogenous peroxidase was blocked with Bloxall (Vector Laboratories Newark, CA, USA; SP-6000-100) and unspecific binding was blocked with 5% normal goat serum for 1 h. Sections were incubated overnight with the following primary antibodies in 2.5% goat serum: Rabbit anti-Ccn2/Ctgf (1:100, Novus Biologicals, Littleton, CO, USA; nb100-724), rabbit anti-Ptgs2 (1:100, Proteintech, San Diego, CA, USA; 12375-1-AP) or rabbit anti-Piezo1 antibody (1:50, Proteintech; 15939-1-AP) overnight at 4 °C. After washing, sections were incubated with Dako EnVision Flex HRP (Agilent Technologies; SM802) as secondary antibody for 30 min. Staining was developed by incubation with the chromogenic substrate 3,3′-Diaminobenzidine (DAB Substrate Kit, Vector Laboratories; SK-4100). Slides were counterstained with Mayer’s Hematoxylin (Sigma-Aldrich, St. Louis, MO, USA; MHS16), blued under running tap water, dehydrated and mounted with DPX mountant (Sigma-Aldrich, 44581). Semi-quantitative scoring of positively stained cells, with a grading system ranging from 0 to 4, where no staining was graded as 0, and strong staining of all cells was graded as 4, was performed independently by three blinded observers. The quantification was performed for the articular cartilage, subchondral bone plate, and trabecular bone of the femoral head, as well as for cartilage, zones of endochondral ossification, bone and fibrosis within the osteophytes. The scores of the observers were then averaged for each compartment.

For immunohistochemistry of mouse lumbar spine sections, lumbar spines from 2-week-old mice were decalcified in 20% EDTA for one week, and sagittal plane paraffin sections were prepared as described above. Deparaffinized sections were treated with pepsin for 60 min at 37 °C (Agilent, SantaClara, CA, USA; S3002), followed by hyaluronidase digestion (0,5%; Sigma-Aldrich; H3506) for 20 min at 37 °C. Peroxidase and serum blocking were performed as described above. Sections were then incubated with the following primary antibodies in 2.5% goat serum: Rabbit anti-collagen type I (1:100, Kerafast, Boston, NE, USA; ENH018-FP), rabbit anti-collagen type II (1:100, Abcam, Cambridge, UK; ab34712) for 1 h at room temperature. Secondary antibody incubation and further processing were performed as described above.

### TRAP staining

For the staining of tartrate-resistant acid phosphatase (TRAP), lumbar spine paraffin sections from two-week-old mice were incubated in a substrate solution (40 mmol/L sodium acetate, 10 mmol/L sodium tartrate, 1.6 mmol/L fast red violet, 700 mmol/L naphthol, pH 5; all chemicals Sigma-Aldrich) for 90 min and counterstained with Mayer’s hematoxylin (Sigma-Aldrich). Quantification was performed using the Osteomeasure (OsteoMetrics, Inc.) system as follows: The hypertrophic zone and underlying primary spongiosa were marked as “bone” and TRAP-positive cells were marked as “osteoclasts”. The parameter number of osteoclasts per bone perimeter (N.Oc/B.Pm) was derived, which represents the number of TRAP-positive cells per tissue perimeter (TRAP^+^ cells/T.Pm in mm^−1^).

### Statistical analysis

Data are shown as box plots with dots representing individual values and indicating the group size. Statistical analysis was performed using SPSS (version 29, IBM, Armonk, USA) and GraphPad Prism (version 9, GraphPad Software, La Jolla, USA) for visualization. For comparison of more than two groups one-way ANOVA (one variable) or two-way ANOVA (two variables) with Tukey post-hoc test was performed. Comparisons between two groups were performed with the Student’s *t* test. All statistical tests were two-sided and a significance threshold of 0.05 was chosen.

### Supplementary information

Supplementary Information

## Data Availability

The data that support the findings of this study are available from the corresponding authors on request. Gene expression datasets from the genome-wide transcriptomic analyses has been deposited at the GEO database of the National Center for Biotechnology Information (NCBI, https://www.ncbi.nlm.nih.gov/geo/) under accession number GSE230071.
