# Supplementary material for: Piezo1 expression in chondrocytes controls endochondral ossification and osteoarthritis development
Source: Bone Res. 2024 Feb 23;12:12. doi: 10.1038/s41413-024-00315-x (PMC10891122; doi:10.1038/s41413-024-00315-x)
Supplement: Supplementary file 1 — Supplementary Information [file 41413_2024_315_MOESM1_ESM.docx]

**Supplementary Information for**

**Piezo1 expression in chondrocytes controls endochondral ossification and osteoarthritis development**

Laura J. Brylka^1*^, Assil-Ramin Alimy^2*^, Miriam E.A. Tschaffon-Müller^3^, Shan Jiang^2^, Tobias Malte Ballhause^2^, Anke Baranowsky^2^, Simon von Kroge^1,2^, Julian Delsmann^2^, Eva Pawlus^1^, Kian Eghbalian^1^, Klaus Püschel^4^, Astrid Schoppa^3^, Melanie Haffner-Luntzer^3^, David J. Beech^5^, Frank Timo Beil^2^, Michael Amling^1^, Johannes Keller^2^, Anita Ignatius^3^, Timur A. Yorgan^1,#^, Tim Rolvien^2,#^, and Thorsten Schinke^1,#^

*LJB and A-RA contributed equally and share first authorship.

^#^TS, TR, and TAY jointly supervised this work and share last authorship.

^1^Department of Osteology and Biomechanics, University Medical Center Hamburg-Eppendorf, 20246, Hamburg, Germany.

^2^Department of Trauma and Orthopedic Surgery, University Medical Center Hamburg-Eppendorf, 20246, Hamburg, Germany.

^3^Institute of Orthopedic Research and Biomechanics, University Medical Center Ulm, Baden-Württemberg, 89081, Ulm, Germany.

^4^Department Legal Medicine, University Medical Center Hamburg-Eppendorf, 20246, Hamburg, Germany.

^5^Leeds Institute of Cardiovascular and Metabolic Medicine, School of Medicine, University of Leeds, LS2 9JT, Leeds, UK.

Corresponding Authors

Dr. Thorsten Schinke ([schinke@uke.de](mailto:schinke@uke.de))

Dr. Tim Rolvien ([t.rolvien@uke.de](mailto:t.rolvien@uke.de))

**
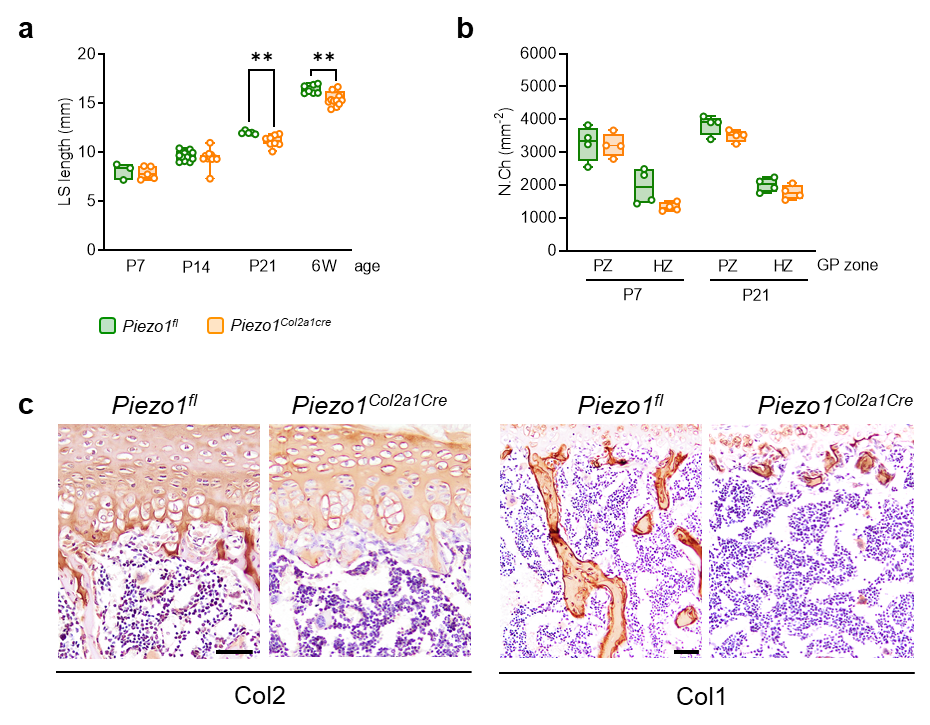
**

**Fig. S1 Inactivation of Piezo1 in chondrocytes specifically reduces trabecular bone structures below the growth plates.**

**a** Lumbar spine (LS) length of *Piezo1^fl^* and *Piezo1^Col2a1Cre^* littermates at the indicated ages. n≥5. **b** Quantification of the number of chondrocytes per mm^2^ in the proliferative (PZ) and hypertrophic zone (HZ) of the growth plate in *Piezo1^fl^* and *Piezo1^Col2a1Cre^* littermates at the ages of P7 and P21. n=4. **c** Representative histological images of lumbar spine sections from 2-week-old mice stained for type II (left) and type I collagen (right) by immunohistochemistry, scale bars: 50 µm. Statistical analysis was conducted by Student’s t-test comparing different genotypes for each age. **p<0.01.


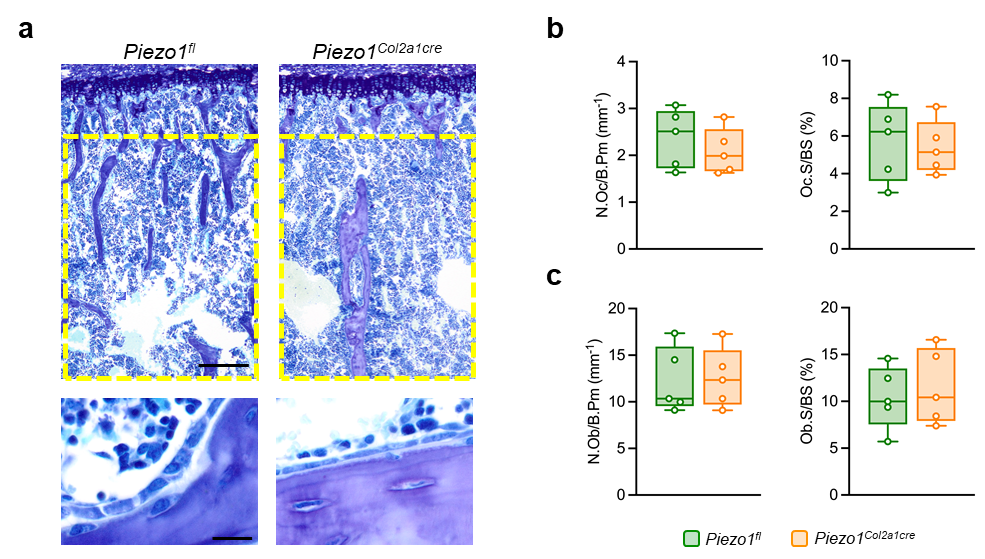


**Fig. S2 Cellular histomorphometry in lumbar spines of 6-week-old mice.**

**a** Toluidine blue-stained acrylate sections of the lumbar spine of *Piezo1^fl^* and *Piezo1^Col2a1Cre^* littermates at 6 weeks of age. The area in which cellular histomorphometry was performed, is highlighted in the upper panels (scale bar: 250 µm). Lower panels show high magnification images of osteoblasts which displayed a flattened appearance in *Piezo1^Col2a1Cre^* mice (scale bar: 10 µm). **b** Histomorphometric quantification of osteoclast number (number of osteoclasts per bone perimeter (N.Oc/B.Pm) and surface per bone surface (Oc.S/BS). **c** Histomorphometric quantification of osteoblast number (number of osteoblasts per bone perimeter (N.Ob/B.Pm) and surface per bone surface (Ob.S/BS).

**Fig. S3 Histology of non-fractured rib bones from 7-day-old *Piezo1^fl^* and *Piezo1^Col2a1Cre^* mice.**

Representative histological sections of ribs from *Piezo1^fl^* and *Piezo1^Col2a1Cre^* littermates at postnatal day 7. For each genotype, the left and top right images show von Kossa-stained sections, middle right panels show toluidine blue-stained sections and lower right panels show Safranin-O-stained sections of the same ribs each. The right panels show a high magnification of the growth plate and subchondral bone area. Scale bars: 100µm (overview) and 50µm (close-up).


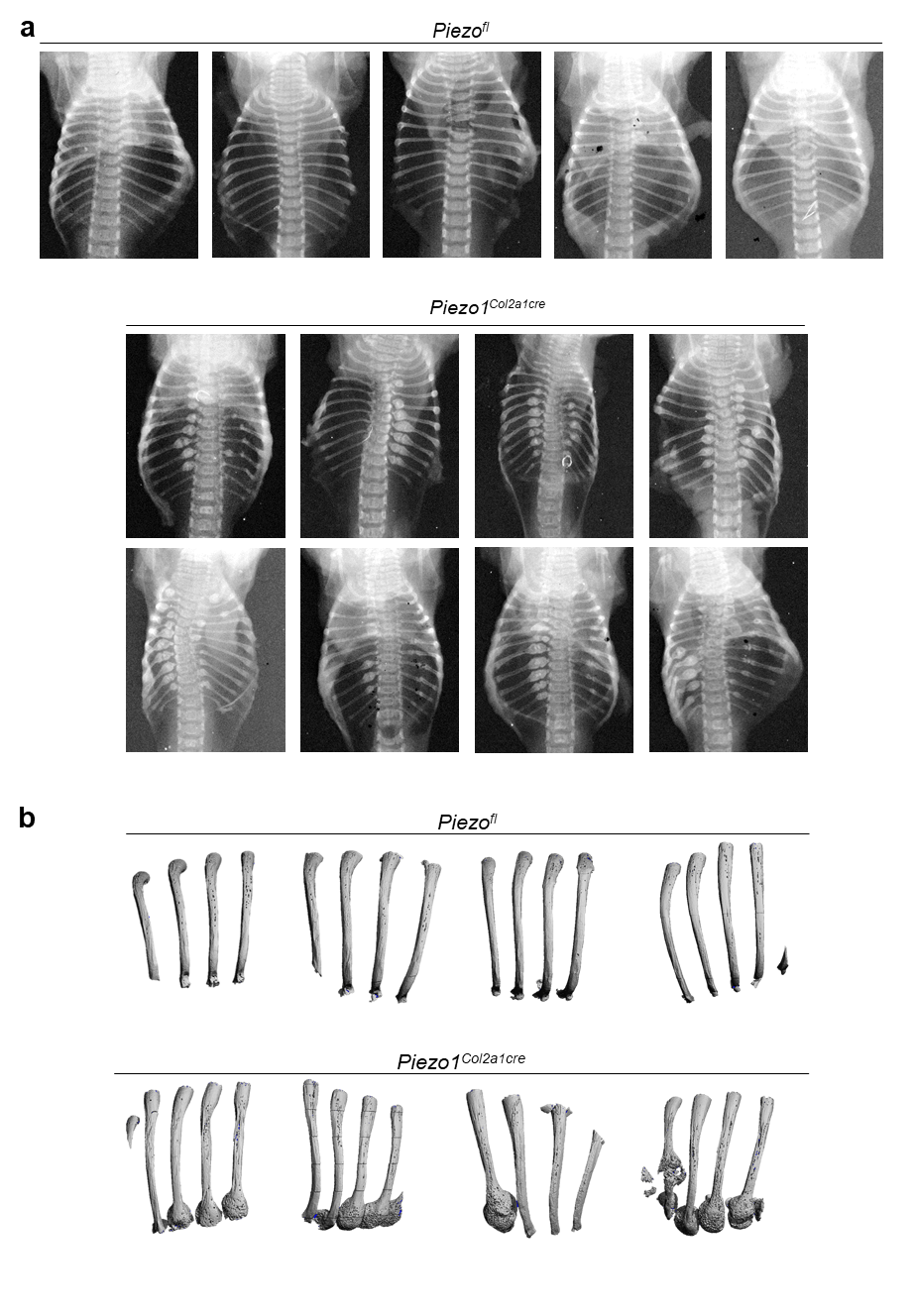


**Fig. S4 Rib fractures in all *Piezo1^Col2a1Cre^* mice at P7.**

**a** Contact radiographs of rib cages from *Piezo1^fl^* and *Piezo1^Col2a1Cre^* littermates at postnatal day 7 (P7) demonstrating that all *Piezo1^Col2a1Cre^* display rib fractures close to the growth plates. **b** Reconstructions of rib µCT scans from *Piezo1^fl^* and *Piezo1^Col2a1Cre^* littermates at P7.


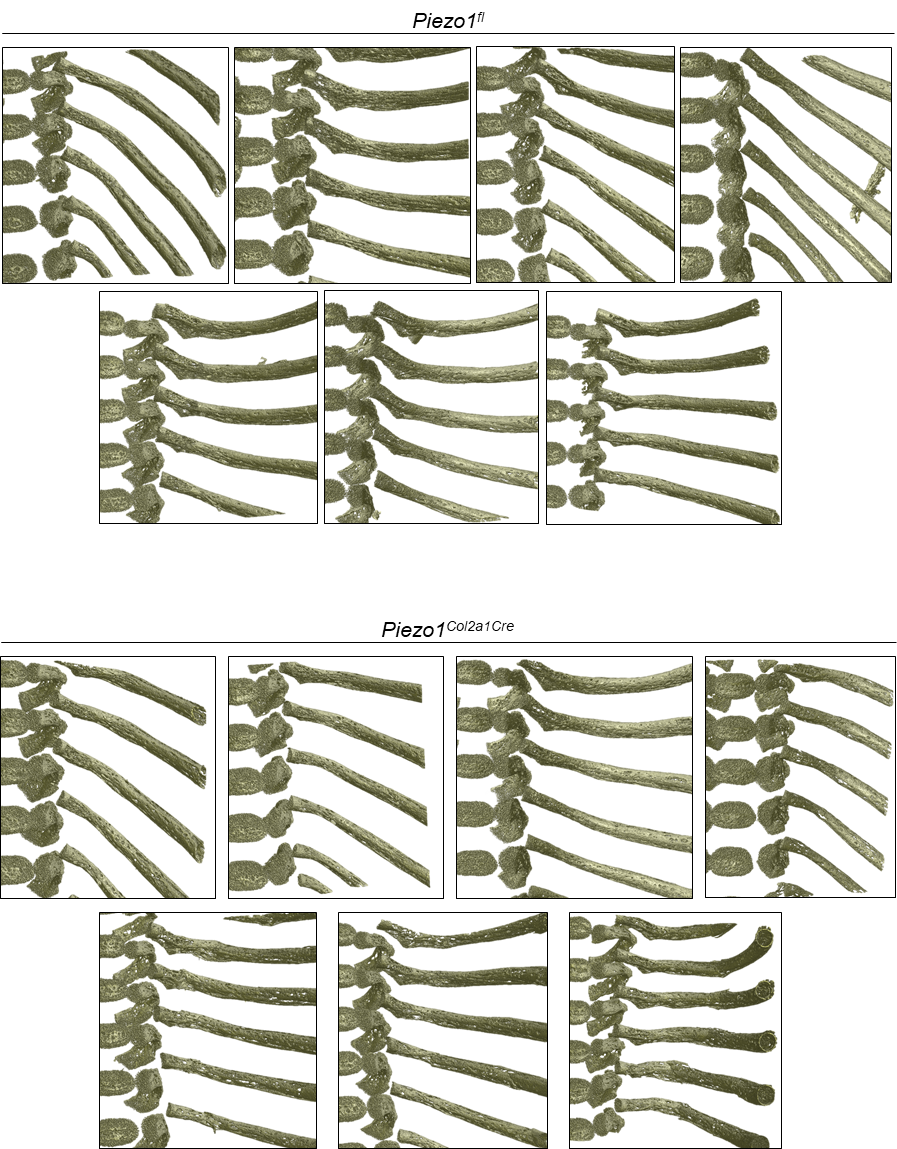


**Fig. S5 µCT-scans showing rib bones of *Piezo1^fl^* and *Piezo1^Col2a1Cre^* mice immediately after birth (P0).**

Reconstructions of rib µCT scans from all analyzed newborn (P0) *Piezo1^fl^* and *Piezo1^Col2a1Cre^* littermates.


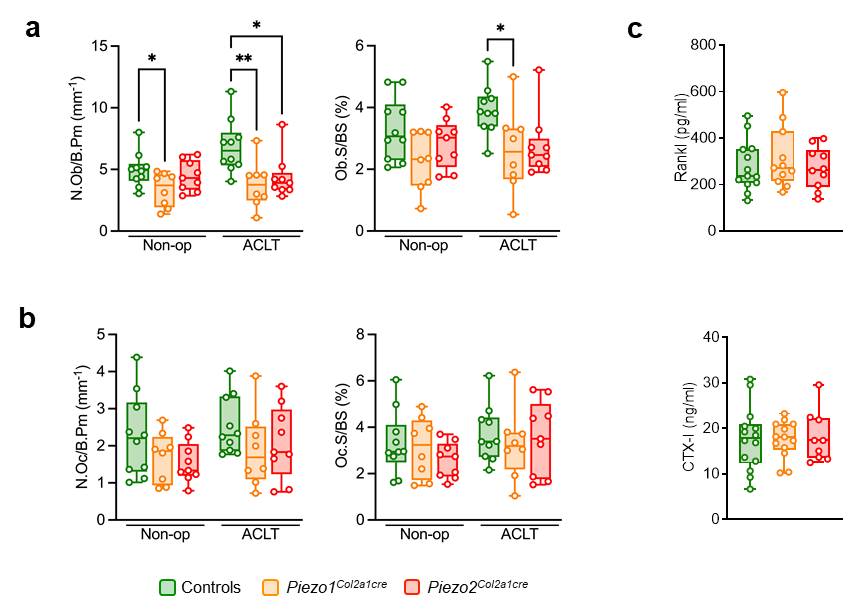


**Fig. S6 Subchondral bone histomorphometry and bone resorption markers in mice that were subjected to ACLT.**

**a** Histomorphometric quantification of osteoblast number (number of osteoblasts per bone perimeter (N.Ob/B.Pm) and surface per bone surface (Ob.S/BS). **b** Histomorphometric quantification of osteoclast number (number of osteoclasts per bone perimeter (N.Oc/B.Pm) and surface per bone surface (Oc.S/BS). **c** Concentrations of Rankl and CTX-I in the serum of ACLT mice 8 weeks after surgery. Statistical analysis was conducted with one-way ANOVA (Tukey). *p<0.05, **p<0.01.


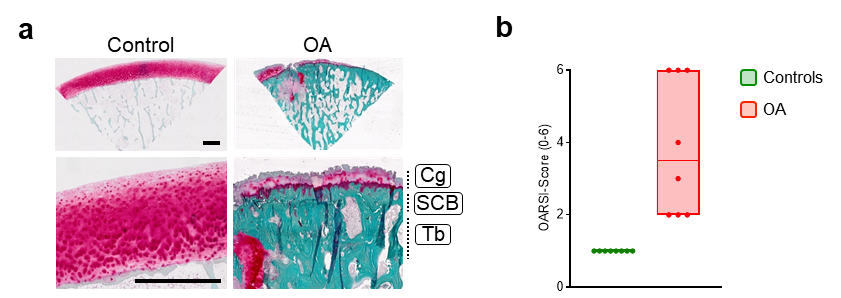


**Fig. S7 Human femoral head specimens.**

**a** Representative Safranin-O-stained sections of the femoral head from control and OA specimens. Top panel: overview; bottom panel: detailed view of the osteochondral unit, scale bar: 2 mm. **b** Quantification of the OARSI score in control and OA specimens of the femoral head.


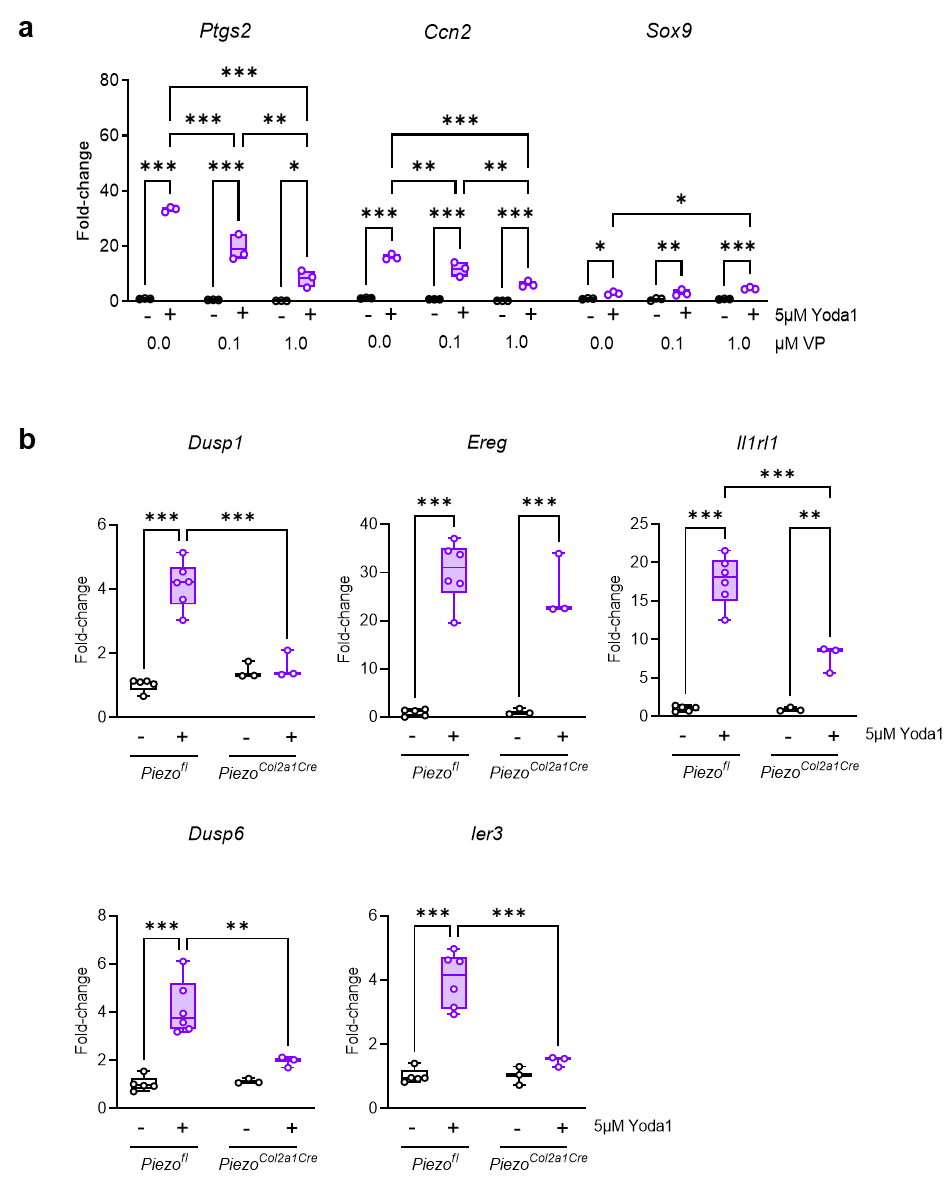


**Fig. S8 Expression analysis of Piezo1-regulated genes**

**a** Quantitative real-time PCR analysis of *Ptgs2*, *Ccn2* and *Sox9* in ATDC5 cells after stimulation with Yoda1 in the presence or absence of Verteporfin at different concentrations. **b** Quantitative real-time PCR analysis of the indicated genes in cultured primary chondrocytes isolated from *Piezo1^fl^* and *Piezo1^Col2a1Cre^* littermates treated with Yoda1 or DMSO. Statistical analysis was conducted with two-way ANOVA (Tukey) *p<0.05, **p<0.01, ***p<0.001.


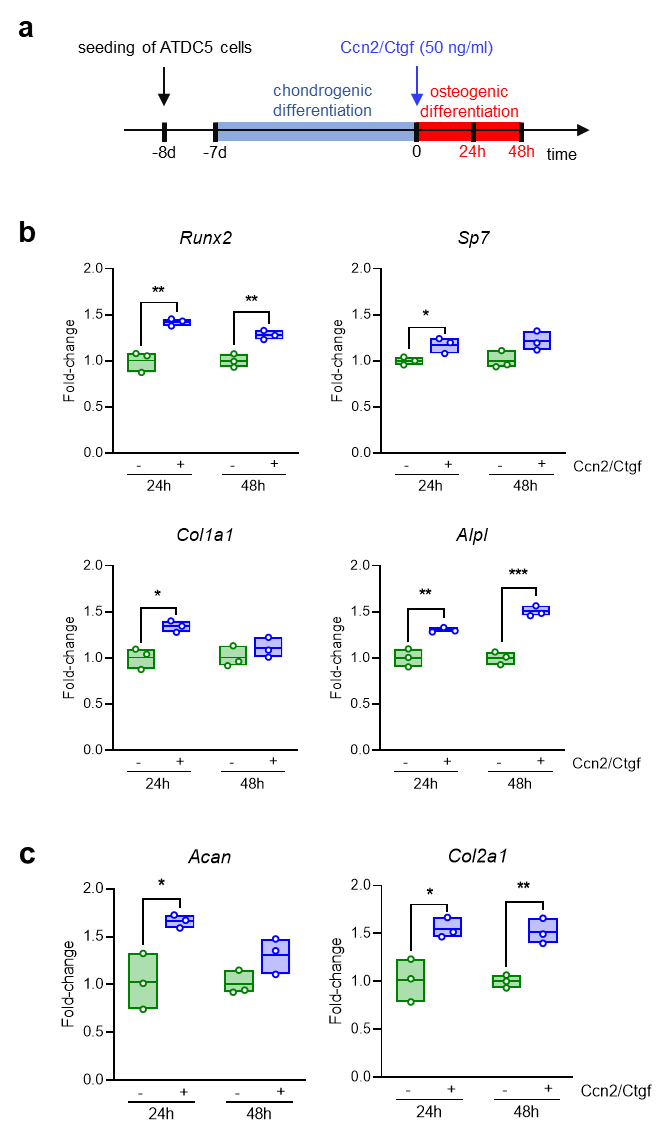


**Fig. S9 Influence of Ccn2/Ctgf on osteogenic gene expression in ATDC5 cells.**

**a** Schematic presentation showing the timeline of the transdifferentiation assay performed with ATDC5 cells. **b** Quantitative real-time PCR analysis of the indicated osteogenic markers 24 and 48 hours after osteogenic induction in the presence and absence of Ccn2/Ctgf. **c** Expression analysis of chondrogenic markers in the same samples. Statistical analysis was conducted with Student’s t-test comparing controls and Ccn2/Ctgf-treated samples for each timepoint. *p<0.05, **p<0.01, ***p<0.001.

**Supplementary Table S1 Primer Sequences**

| Gene name | forward primer sequence (5' to 3') | reverse primer sequence (3' to 5') |
| --- | --- | --- |
| *Acan* | AACTTCTTTGCCACCGGAGA | GGTGCCCTTTTTACACGTGAA |
| *Alpl* | GCTGATCATTCCCACGTTTT | GAGCCAGACCAAAGATGGAG |
| *B2m* | ATACGCCTGCAGAGTTAAGCA | TCACATGTCTCGATCCCAGT |
| *Col1a1* | GAGCGGAGAGTACTGGATCG | GTTCGGGCTGATGTACCAGT |
| *Col2a1* | CCTGTCTGCTTCTTGTAAAAC | TGGGTATCATCAGGTCAGGT |
| *Runx2* | CCACCACTCACTACCACACG | CACTCTGGCTTTGGGAAGAG |
| *Sp7* | CCTTAACCCAGCTCCCTACC | ACCGCCTTGGGCTTATAGAC |
